# Laminar organization of the anterior olfactory nucleus—the interplay between neurogenesis timing and neuroblast migration

**DOI:** 10.3389/fnins.2025.1546397

**Published:** 2025-04-30

**Authors:** Eduardo Martin-Lopez, Bowen Brennan, Marion Lefèvre, Natalie J. Spence, Kimberly Han, Charles A. Greer

**Affiliations:** ^1^Department of Neurosurgery, Yale University School of Medicine, New Haven, CT, United States; ^2^Department of Neuroscience, Yale University School of Medicine, New Haven, CT, United States; ^3^Interdepartmental Neuroscience Program, Yale University School of Medicine, New Haven, CT, United States

**Keywords:** AON, neurogenesis, development, electroporation, thymidine analogs, radial glia

## Abstract

**Introduction:**

The anterior olfactory nucleus (AON) is a laminar structure embedded within the olfactory peduncle which serves as the conduit for connectivity between the olfactory bulb (OB) and the central processing centers of the brain. The largest portion of the AON is a ring of neurons and fibers that surround the core of the peduncle, the *pars principalis* (AONpP). The AONpP is further subdivided into an outer plexiform layer, or layer 1 (L1), that contains axons and dendrites, and an inner cell zone, or layer 2 (L2), formed by densely packed pyramidal cells. Relative to other regions of the olfactory system, the development of the AON remains poorly understood.

**Methods:**

We performed injections of thymidine analogs in pregnant mice from E10 to E18 to determine the timeline of AON neurogenesis and used immunohistochemistry to study neuronal phenotypes both at adult and embryonic stages. To better understand migration and differentiation of the AON neurons, we labeled AON precursors using *in utero* electroporations with the piggyBac transposon into the rostral lateral ganglionic eminence, the embryonic source of AON neurons.

**Results:**

Our analyses established that the earliest neurons targeted to the AON laminae arose at E10 with neurogenesis peaking at E13. In L1, we found a caudal-to-rostral neurogenic gradient not detected in L2. Quantification across the cardinal axes showed no gradients in L2 and a medial-to-lateral gradient for L1. Using immunohistochemistry, we found that AON neurons express the most common cortical markers Tbr1, Ctip2, NeuroD1, Sox5 and Cux1+2 at adult stages without laminar distinction. Tbr1 and NeuroD1 first appeared embryonically at E12, while Ctip2 and Sox5 were present at E13, following a dorsal-ventral pattern. Cux1+2 was not detected embryonically. Embryonically, our data on neuroblasts migration revealed that AON neuroblasts use a scaffold of radial glia to migrate to their final destinations in both L1 and L2 through a caudal-to-rostral migratory gradient.

**Conclusion:**

For the first time, our data show a comprehensive timeline for the AON neurogenesis across the anatomical axes, and a detailed analysis on neuroblast migration in the mouse embryo. These data are crucial to understanding the embryonic formation and relationship of relay stations along the olfactory pathway.

## Introduction

In the olfactory pathway, the anterior olfactory nucleus (AON) composes the majority of the olfactory peduncle and serves as a relay station between the olfactory bulb (OB) and the tandem piriform cortex (PC)—tubular striatum [TuS; also known as the olfactory tubercle (Wesson, 2020)], as well as other non-olfactory areas of the brain (Brunjes et al., 2005; Brunjes et al., 2011; Brunert et al., 2023). Despite the label “nucleus,” there is a current consensus that the AON is a cortical structure (Haberly, 2001), yet we will continue to retain AON nomenclature, “*in deference to historical and literary convention*” (Brunjes et al., 2005). The specific functions of the AON are still under investigation, but multiple studies have determined its involvement in memory formation and learning behaviors related to olfaction through its extensive connectivity with other parts of the brain, particularly the limbic system (Hamrick et al., 1993; Levinson et al., 2020; Quintela et al., 2022; Brunert et al., 2023). Among the AON circuits, two main loops control how the olfactory information is filtered between the OB and PC (Brunjes et al., 2005; Chae et al., 2022). The first loop is the direct excitation of AON neurons from the OB mitral and tufted cells (M/Tc) via the lateral olfactory tract (LOT) (Shipley and Ennis, 1996; Nagayama et al., 2010). In return, AON neurons send centrifugal projections, both ipsi- and contra-laterally, to the OB via the anterior commissure (AC) to excite granule cells (Daval and Leveteau, 1974; Imai and Sakano, 2008). These centrifugal projections generate an indirect inhibition of M/Tc (Medinaceli Quintela et al., 2020). The second loop is the feedforward direct stimulation of neuronal activity in the PC (Russo et al., 2020).

Anatomically, the AON is a bilaminar structure that encircles the central core of the olfactory peduncle. The central core consists of the migratory neuroblasts that form the rostral migratory stream and axon bundles of the AC and the medial forebrain bundle (Whitman and Greer, 2009; Brunjes et al., 2011). Due to its proximity with the OB and PC, the anatomy of the AON changes along its rostral-to-caudal axis. The most rostral part of the AON is formed by a band of densely packed cells connected to the caudal-lateral section of the OB, known as *pars externa* (AONpE) (Brunjes et al., 2005). The AONpE is responsible for the projections to the contralateral OB (Reyher et al., 1988; Yan et al., 2008). Continuing caudally, the AON becomes the characteristic ring of cells and fibers that form most of the olfactory peduncle known as *pars principalis* (AONpP) (Brunjes et al., 2005; Brunjes and Osterberg, 2015). The AONpP is formed by two laminae: (1) an outer plexiform layer (*opl*) or layer 1 (L1); and (2) an inner cell zone (*icz*) or layer 2 (L2). L1 is composed of two sublayers: (1) a superficial region formed by the LOT axon terminals intermingled with the distal tips of the apical dendrites from the AON principal neurons or L1a; and (2) a deeper region formed by apical dendrites and cortico-cortical association axons or L1b. In contrast, L2 is formed by a plethora of different neuronal types exhibiting a variety of morphologies and expressing distinct markers typical of projection and inter-neurons (Reyher et al., 1988; Valverde et al., 1989; Kay and Brunjes, 2014; Brunjes and Osterberg, 2015; Brunert et al., 2023). Structurally, the AONpP has been arbitrarily subdivided along the cardinal planes into *pars dorsalis*, *pars medialis*, *pars lateralis,* and *pars ventralis*, which represent areas that project to different regions of the olfactory system. For example, the ipsilateral OB receives projections from *pars medialis*, while the feedforward projections to PC are from *pars dorsalis and lateralis* (Herrick, 1924; Brunjes et al., 2005).

Developmentally, AON neurons seem to follow neurogenic gradients that are intermediate to those of projection neurons from the OB and PC (Hinds, 1968; Imamura et al., 2011; Martin-Lopez et al., 2019a). Early studies using ^3^H-thymidine suggested some discrepant data on AON neurogenesis along the different cardinal planes. The peak for earliest generation of AON neurons in mouse was detected in the *pars lateralis and pars posterior* (Creps, 1974), while Hinds found the *pars externa and medialis* to be the earliest (Hinds, 1967). Alternatively, it has been shown in rats that *pars medialis* and *ventralis* generate earlier, with all *pars* subdivisions following a caudal-to-rostral neurogenic gradient (Bayer, 1986a). These differences highlight the importance of carefully investigating neurogenesis between the different species of animals as well as revisiting these analyses using contemporary methods.

To resolve the discrepancies in our understanding of AON development, in this work we studied AON neurogenesis by injecting mice with analogs of thymidine from embryonic day 10 (E10) to E18, to cover the entire timeline of neuronal differentiation from postmitotic to the last gestational day (Bulfone et al., 1995; Greig et al., 2013). We then compared neurogenesis along the rostro-caudal axis and across the different cardinal planes as described in rats (Bayer, 1986a) to study neurogenic gradients in mice. We characterized the phenotypes of AON neurons at adult stages using markers specific for different layers of the neo- and paleo- cortex and their onset embryonically. In addition, we used contemporary techniques based on the piggyBac transposon and *in utero* electroporations (IUE) to elucidate the migratory pathways, neuronal differentiation, and maturation of AON neuroblasts (Martin-Lopez et al., 2019a). The IUE was targeted in the most rostral end of the lateral ganglionic eminence (rLGE), the origin of AON neurons (Puelles et al., 2000; Garcia-Moreno et al., 2008).

## Materials and methods

### Animals

All experiments were performed using male and female CD1 mice derived from pregnant females from Charles River (cd-1r-igs). Adult mice were used to assess the morphology and neuronal characterization of the AON. Pregnant females were used for IUE and their progeny to assess neurogenesis and neuronal characterization of the AON through development at different embryonic stages. In analyzing embryonic timepoints, embryonic day 0 (E0) was considered as the day of the vaginal plug. Mice were maintained on a 12 h light cycle in the vivarium at Yale University. All protocols and procedures were approved by Yale University Animal Care and Use Committee.

### Sample size calculations and power analysis

The minimum size (n) for each experiment was calculated by performing prior power analyses using the G*Power 3.1.9.7 software. For layer thickness calculations, we made assumptions based on data from our previous publication in anterior PC (Martin-Lopez et al., 2019a). We analyzed four groups (LOT, Ia, Ib and II), and used the Cohen’s f approximation to calculate an effect size of *f* = 5.2. By establishing an *α* = 0.05 and a power of 95%, we obtained a total sample size of 8 (2 mice per group) with an actual power of 0.99. For neurogenesis analysis, we performed a pilot study using 3 mice per group and quantified the number of cells expressing analogs of thymidine in L1 and L2 at each embryonic age (E10-18; nine groups). Using an *α* = 0.05 and a power of 95%, we obtained an effect size of 1.3 for L1, and 2.2 for L2. The total sample size was 27 (3 mice per group) and 18 (2 mice per group) for L1 and L2, respectively. The “actual power” calculated in G*Power for neurogenesis analysis was 0.99 for both layers. The final number of animals we used in each study was higher and indicated below for each experiment.

Since we were interested in neurogenesis of the AON from an exclusively anatomical point of view, one that did not involve any external manipulation or behavioral assessment, we anticipated that the litter effect was not significant between the groups. Previous work demonstrates that, at least anatomically, brain variability among different CD1 mice is insignificant, and can be used to reduce the number of animals per study while still producing reliable and reproducible results (Scholz et al., 2016). The litter effect becomes an issue only to those studies that analyze behavioral changes with or without interventions on the mice, as well as in neurodevelopmental disorders (Jimenez and Zylka, 2021; Valiquette et al., 2023). In most cases using mice from different litters seek to increase the statistical power and reproducibility (see Figure 4 from Lazic and Essioux, 2013), but Lazic and Essioux also agree that using many litters may represent ethical concerns regarding the amount of animals used on each study, and they also state that: *“Whether litter is an important factor for any particular outcome is then an empirical question, and if it is not important then it need not be included in the analysis”* (Lazic and Essioux, 2013). Therefore, since we used CD1 mice for our analysis at P21 to make anatomical studies on neurogenesis, we did not perform any behavioral assessment, and because we obtained a high statistical power from our preliminary data, we concluded that using littermates for our analysis did not impact the conclusions of this study.

### Thymidine analogs injection

Thymidine analogs were used to quantify neurogenesis in the AON. We used a protocol that involved the injection of two different thymidine analogs that can be detected separately by using specific primary antibodies: 5-Iodo-2′-deoxyuridine (IdU; Sigma Aldrich, I7125) followed by 5-Chloro-2′-deoxyuridine (CldU; Sigma-Aldrich, C6891), in the same pregnant female but at different embryonic stages, so we could track two embryonic ages per dam (five pregnant females total) (Martin-Lopez et al., 2019a). Double injections of 50 mg/kg of these analogs, separated 2 h apart, were injected intraperitoneally (IP) into four pregnant dams with the following embryonic sequence: E10 + E14, E11 + E15, E12 + E16, E13 + E17, and one pregnant female only at E18 (IdU). For the analyses, 6 mice/timepoint (*n* = 6: 3 males +3 females) were euthanized with an overdose of Euthasol (Covetrus) on postnatal day 21 (P21), and then transcardially perfused with cooled 4% paraformaldehyde (PFA) in phosphate-buffered saline (PBS). Brains were dissected from the skulls and postfixed for 24 h in PFA before cryoprotection in 30% sucrose-PBS. After sinking in the sucrose solution, brains were embedded in Tissue-Tek OCT compound (Fisher Scientific, Cat#4585) before being rapidly frozen for cryo-sectioning.

### *In utero* injection and electroporation (IUE)

To study the migration and differentiation of neuroblasts during AON development, we labeled progenitor cells at the dorsal, most rostral portion of the lateral ganglionic eminence (rLGE), previously identified as the neurogenic region for AON neurons (Garcia-Moreno et al., 2008; Huilgol and Tole, 2016). Prior to surgery, we prepared a suspension of two piggyBac transposon plasmids in ddH_2_O both at a concentration of 1 μg/μL: pPB-CAG-EGFP and pCAG-PBase, supplemented with 0.05% of fast green (Sigma-Aldrich, F7252) as previously described (Martin-Lopez et al., 2019a). Then, pregnant females at E11 (10 total, 2 per group) were anesthetized with 2.0% isoflurane (Covetrus) and placed on their backs on a dissecting board. Their abdominal cavities were exposed by performing an incision in the midline of the skin and along the alba line of the peritoneal membrane. Embryos from the abdomen were placed onto a sterile gauze that was pre-humidified with pre-warmed lactated ringer, and each embryo was individually injected with the plasmid solution inside the lateral ventricles using borosilicate capillaries connected to a Picospritzer (General Valve Corporation). Plasmids were electroporated by delivering 5 pulses of 35 V using a pair of gold tweezers (Genepaddles-542, Harvard Apparatus, 45-0122) connected to an ECM 830 electroporator (BTX Harvard Apparatus). Finally, embryos were returned to the abdomen, and the abdominal cavity was filled with pre-warmed lactated ringer prior to suturing the peritoneal membrane with 5/0 PGA absorbable sutures (AD Surgical, S-G518R13-U), and the skin with 5/0 silk braided sutures (AD Surgical, S-S518R13). Postsurgical care involved the administration of 4 mg/kg of the analgesic Meloxicam (Covetrus, 049756) injected subcutaneously for 48 h. Embryos were collected from the E12 to E17 stages. The pregnant females were euthanized with an overdose of CO_2_, their abdomens opened to expose the uterus, and the embryos extracted from the yolk sac to proceed for a rapid brain extraction before fixing by immersion in 4% PFA for 48 h. After fixation, brains were checked for positive IUE into the AON, cryoprotected in 30% sucrose-PBS and tissues embedded in Tissue-Tek OCT compound to prepare blocks for cryosectioning.

### Tissue processing and immunostaining

All adult and embryonic brains were serially sectioned with a Reichert Frigocut cryostat (E-2800) at 20 μm in the coronal plane and collected onto Fisherband ColorFrost Plus slides (Fisher Scientific) Sections were dried on a slide warmer at 50°C and then stored at -80°C until immunohistochemical (IHC) staining. On the day of IHC, all sections were first thawed on the slide warmer at 60°C for 20 min and then washed with PBS to remove the OCT compound.

To detect thymidine analogs, sections were pre-treated for 30 min in 0.025 M HCl at 65°C to denaturalize the DNA and then rinsed with 0.1 M borate buffer (pH 8.5) for 10 min to neutralize the acid. In all other adult and embryonic sections that did not require thymidine analogs exposure, the HCl treatment was replaced by a step that involved incubating the slides for 35 min in 0.01 M citrate buffer (pH 6.0) pre-heated at 65°C, followed by immersion in the same ice-cooled citrate buffer for 5 min. This incubation ensured an antigen unmasking to improve primary antibody recognition in the tissues. After this step, slides were placed horizontally into a humid chamber and unspecific binding of antibodies was blocked by incubating the sections with PBS + 0.1% Triton X100 (PBST) supplemented with 5% Normal Goat Serum (NGS, Accurate Chemicals) and 0.1% Bovine Serum Albumin (BSA, Sigma Aldrich) for 1 h at room temperature (RT). Then, primary antibodies (Table 1) were incubated overnight at 4°C in the humid chamber and washed three times by 10 min. Each with PBST before incubation with specific secondary antibodies (Table 1) for 2 h at RT. The secondary antibody solution was supplemented with 1 μg/mL of DAPI (Sigma-Aldrich, D9542) for nuclear counterstaining.

**Table 1:** Primary and secondary antibodies.

Antigen	Primary Ab	Source (Cat. #)	RRID	Dilution	Secondary Ab	Source	Dilution
BrdU/IdU	Mouse IgG1	BD Biosciences (347580)	AB_400326	1:200	Goat α-mouse IgG (H + L)-Alexa 555 Superclonal™ Recombinant	Thermo Fisher Scientific	1:1,000
BrdU/CldU	Rat IgG2a	Abcam (ab6326)	AB_305426	1:300	Goat α-rat IgG Alexa 488	Thermo Fisher Scientific	1:1,000
Calretinin	Rabbit clone SP13	Abcam (ab16694)	AB_2259432	1:500	Goat α-rabbit IgG (Heavychain) Superclonal Recombinant Alexa 488	Thermo Fisher Scientific	1:1,000
Ctip2	Rat IgG2a clone 25B6	Abcam (ab18465)	AB_2064130	1:500	Goat α-rat IgG Alexa 546	Thermo Fisher Scientific	1:1,000
Cux1 + Cux2	Rabbit clone [EPR26509-154]	Abcam (ab309139)	AB_3094470	1:50	Goat α-rabbit IgG (Heavychain) Superclonal Recombinant Alexa 488	Thermo Fisher Scientific	1:1,000
GFAP	Rabbit IgG	Biolegend (840001)	AB_2565444	1:500	Goat α-rabbit IgG Alexa 647	Thermo Fisher Scientific	1:1,000
IBA1	Rabbit IgG	Wako (016–200,001)	AB_839506	1:500	Goat α-rabbit IgG Alexa 647	Thermo Fisher Scientific	1:1,000
Map2	Chicken Poly.	EMD Millipore (AB5543)	AB_571049	1:500	Goat α-chicken IgY Alexa 555	Thermo Fisher Scientific	1:1,000
Myelin basic protein (MBP)	Rat IgG2a clone 12	Novus Biologicals (nb600-717)	AB_2139899	1:500	Goat α-rat IgG Alexa 488	Thermo Fisher Scientific	1:1,000
NeuroD1	Mouse IgG2a	Abcam (ab60704)	AB_943491	1:500	Goat α-mouse IgG (H + L)-Alexa 555 SuperclonalTM Recombinant	Thermo Fisher Scientific	1:1,000
Sox5	Rabbit Poly.	Abcam (ab94396)	AB_10859923	1:250	Goat α-rabbit IgG (Heavychain) Superclonal Recombinant Alexa 488	Thermo Fisher Scientific	1:1,000
Tbr1	Rabbit Poly.	Abcam (ab31940)	AB_2200219	1:500	Goat α-rabbit IgG (Heavychain) Superclonal Recombinant Alexa 488/Goat *α*-rabbit IgG Alexa 546	Thermo Fisher Scientific	1:1,000
RC2	Mouse IgM λ light chain	Dev. Studies Hybriodoma Bank (RC2)	AB-531887	1:40	Goat α-mouse IgM (Mu Chain); Streptavidin-Alexa 647	Vector; BioLegend	1:1,000

### Imaging and AON nomenclature

In order to be consistent with convention, in the remainder of this article we will use Roman numerals to label the layers of the neocortex and PC while the layers of the AON will be labeled with Arabic numerals.

Images for quantification of thymidine analogs were acquired using a BX51 Olympus epifluorescence microscope. All other images were taken using a laser scanning confocal microscope (Zeiss LSM 800 with Airyscan). For all statistical analysis we used the software GrapPad Prism 10.4.0 and all data in graphics is represented as mean ± SEM. Statistically significant comparisons are summarized in Table 2.

**Table 2:** Statistical analysis with significances.

Analysis (figure)	Test	Pairwise comparisons	Statistical value	Adjusted *p*-value
Layer thicknesses (Figure 1)	One-way ANOVA + Tukey’s multiple-comparison test	LOT vs. L1b	Mean Diff. 48.98/ 95.00% CI of diff. 8.433 to 89.52	0.0170
		L1a vs. L2	Mean Diff. −77.85/ 95.00% CI of diff. −118.4 to −37.31	0.0005
		L1b vs. L2	Mean Diff. −88.24/ 95.00% CI of diff. −128.8 to −47.69	0.0002
Neurogenesis in L1 (Figure 3)	One-way ANOVA + Tukey’s multiple-comparison test	E10 vs. E11	Mean Diff. −88.26/ 95.00% CI of diff. −140.8 to −35.70	<0.0001
		E13 vs. E14	Mean Diff. 85.09/ 95.00% CI of diff. 32.54 to 137.6	0.0001
Neurogenesis in L2 (Figure 3)	One-way ANOVA + Tukey’s multiple-comparison test	E11 vs. E12	Mean Diff. −137.9/ 95.00% CI of diff. −229.3 to −46.53	0.0004
		E13 vs. E14	Mean Diff. 165.1/ 95.00% CI of diff. 73.67 to 256.5	<0.0001
Neurogenesis along the cardinal axes (rostral plane) in L1 (Figure 4C: Rostral)	Two-way ANOVA + Tukey’s multiple-comparison test	E12 v E13	Mean Diff: −456.5/ 95.00% CI of diff. −651.4 to −261.7	<0.0001
		E13 vs. E14	Mean Diff. 597.5/ 95.00% CI pf diff. 387.0 to 808.0	<0.0001
Neurogenesis along the cardinal axes (rostral plane) in L2 (Figure 4C: Rostral)	Two-way ANOVA + Tukey’s multiple-comparison test	E12 v E13	Mean Diff: −226.1/ 95.00% CI of diff. −420.9 to −31.22	0.0132
		E13 vs. E14	Mean Diff: 379.0/ 95.00% CI of diff. 168.5 to 589.5	<0.0001
Neurogenesis along the cardinal axes (medial plane) in L1 (Figure 4C: Medial)	Two-way ANOVA + Tukey’s multiple-comparison test	E10 vs. E13	Mean Diff: −159.5/ 95.00% CI of diff. −306.3 to −12.69	0.0247
		E13 vs. E14	Mean Diff: 170.0/ 95.00% CI of diff. 23.13 to 316.8	0.0134
Neurogenesis along the cardinal axes (medial plane) in L2 (Figure 4C: Medial)	Two-way ANOVA + Tukey’s multiple-comparison test	E11 vs. E12	Mean Diff: −176.7/ 95.00% CI of diff. −312.6 to −40.79	0.0036
		E12 vs. E13	Mean Diff: −182.7/ 95.00% CI of diff. −318.7 to −46.81	0.0024
		E13 vs. E14	Mean Diff: 280.0/ 95.00% CI of diff. 133.2 to 426.8	<0.0001
Neurogenesis along the cardinal axes (caudal plane) in L2 (Figure 4C: Caudal)	Two-way ANOVA + Tukey’s multiple-comparison test	E10 vs. E11	Mean Diff: −202.7/ 95.00% CI of diff. −322.1 to −83.34	0.001
	Two-way ANOVA + Tukey’s multiple-comparison test	E11 vs. E12	Mean Diff: 156.6/ 95.00% CI of diff. 37.23 to 276.0	0.0038
Neurogenesis along the cardinal axes (caudal plane) in L2 (Figure 4C: Caudal)	Two-way ANOVA + Tukey’s multiple-comparison test	E11 vs. E12	Mean Diff: −173.0/ 95.00% CI of diff. −292.4 to −53.67	0.0011
		E12 vs. E13	Mean Diff: −187.7/ 95.00% CI of diff. −307.1 to −68.33	0.0004
		E13 vs. E14	Mean Diff: 316.1/ 95.00% CI of diff. 196.7 to 435.4	<0.0001
Neurogenesis within pars Lateralis in L1 (Figure 4D: Lateralis)	Two-way ANOVA + Tukey’s multiple-comparison test	E12 vs. E13	Mean Diff: −106.3/ 95.00% CI of diff. −206.9 to −5.638	0.0318
		E13 vs. E14	Mean Diff: 236.1/ 95.00% CI of diff. 127.4 to 344.8	<0.0001
Neurogenesis within pars Lateralis in L2 (Figure 4D: Lateralis)	Two-way ANOVA + Tukey’s multiple-comparison test	E11 vs. E12	Mean Diff: −154.0/ 95.00% CI of diff. −254.6 to −53.31	0.0004
		E12 vs. E13	Mean Diff: −182.0/ 95.00% CI of diff. −282.7 to −81.38	<0.0001
		E13 vs. E14	Mean Diff: 396.5/ 95.00% CI of diff. 287.8 to 505.2	<0.0001
Neurogenesis within pars Dorsalis in L2 (Figure 4D: Dorsalis)	Two-way ANOVA + Tukey’s multiple-comparison test	E12 vs. E13	Mean Diff: −290.9/ 95.00% CI of diff. -436.3 to −145.6	<0.0001
		E13 vs. E14	Mean Diff: 343.3/ 95.00% CI of diff. 186.3 to 500.3	<0.0001
Neurogenesis within pars Medialis in L1 (Figure 4D: Medialis)	Two-way ANOVA + Tukey’s multiple-comparison test	E10 vs. E11	Mean Diff: −181.5/ 95.00% CI of diff. -283.9 to −79.16	<0.0001
		E11 vs. E12	Mean Diff: 107.5/ 95.00% CI of diff. 5.104 to 209.8	0.0341
Neurogenesis within pars Medialis in L2 (Figure 4D: Medialis)	Two-way ANOVA + Tukey’s multiple-comparison test	E11 vs. E12	Mean Diff: −171.5/ 95.00% CI of diff. −273.9 to −69.19	0.0001
		E12 vs. E13	Mean Diff: −161.6/ 95.00% CI of diff. −264.0 to −59.25	0.0003
		E13 vs. E14	Mean Diff: 276.9/ 95.00% CI of diff. 174.6 to 379.3	<0.0001
Neurogenesis within pars Ventralis in L1 (Figure 4D: Ventralis)	Two-way ANOVA + Tukey’s multiple-comparison test	E10 vs. E12	Mean Diff: −389.9/ 95.00% CI of diff. −618.1 to −161.7	<0.0001
		E13 vs. E14	Mean Diff: 409.0/ 95.00% CI of diff. 180.8 to 637.2	<0.0001
Neurogenesis within pars Ventralis in L2 (Figure 4D: Ventralis)	Two-way ANOVA + Tukey’s multiple-comparison test	E12 vs. E13	Mean Diff: −312.2/ 95.00% CI of diff. −540.5 to −84.03	0.0020
		E13 vs. E14	Mean Diff: 331.2/ 95.00% CI of diff. 103.0 to 559.4	0.0009

### Quantifications and statistical analysis

To determine the thickness of the laminae of the AON, we measured the length of each layer in the lateral region of anatomically matched confocal images acquired with a 20X objective of a medial plane along the rostro-caudal axis of the AON. The lengths of the laminae were measured with the straight-line tool in ImageJ software on three independent sample images taken in four different adult CD1 mice (postnatal day 21) (*n* = 4: 2 males + 2 females). Layers were identified based upon their specific staining the LOT exclusively expressed CR (green); L1a co-expressed CR and Map2 (yellow); L1b exclusively expressed Map2 (red); and L2 was determined by the presence of densely packed neurons counterstained with DAPI (blue) and Map2 (red) (Sarma et al., 2011; Martin-Lopez et al., 2019a). Layer size was statistically compared by applying a one-way ANOVA followed by a Tukey *post-hoc* test.

To evaluate the AON neurogenesis by embryonic age, we quantified the number of IdU^+^ (Red) and CldU^+^ (Green) cells per mouse throughout the entire AON at each embryonic stage (E10-E18). Three males and three females were used for quantifications at each age. To assess sexual differences, data from males and females were split and compared between age and layer using a one-way ANOVA (Supplementary Figure 1). After finding no sex differences, data from males and females were collapsed into one group for a total of 6 animals (*n* = 6). Three sections per animal were randomly chosen at an intermediate region of the AON along the rostro-caudal axis and cells counted separately in L1 and L2. Cell counts were compared using a one-way ANOVA and normalized and expressed as cells per mm^2^. To analyze neurogenesis along the rostro-caudal axis, four anatomical planes per mouse were captured using a 10X objective and used for quantification. The most rostral plane was used as representative of the “rostral AON,” the two medial planes as the “intermediate AON,” and the most caudal as representative of the “caudal AON.” Data per age were compared by individual layer using a one-way ANOVA. Finally, we studied neurogenesis among subsections representative of the *pars lateralis*, *pars dorsalis*, *pars ventralis*, and *pars medialis* according to their location within the AONpP. Subsection areas were individually determined with the polygon-selection tool in ImageJ for each image. IdU^+^ (Red) and CldU^+^ (Green) cells were manually counted using the ImageJ Cell Counter tool. Quantifications were performed within the AON subsections present at each rostro-caudal plane for the targeted embryonic stage (E10-E17). Four pups (both male and female; *n* = 4) per pregnant dame were used for quantification to assess neurogenesis along the rostro-caudal axis and within the AONpP subsections. The cell counts were normalized and expressed as cells per mm^2^. Statistical differences were determined by applying an ordinary one-way ANOVA followed by a Tukey *post-hoc* test.

Using tissues from E13 to E17 embryos, we estimated the number of cells that showed a tangential (multipolar) or radial (bipolar) migration by studying the morphology of the neuroblasts labeled with the piggyBac transposon. Similarly, we quantified number of IUE^+^ cells that expressed Tbr1, Ctip2, or both. Electroporation fields among embryos were heterogeneous, so that quantifications were made on equivalent sections from 2 to 3 embryos at different ages. Data are presented as percentage of each cell type from the total pool of IUE^+^ cells.

## Results

### Anatomy and neuronal phenotype characterization of the AON *pars principalis* (AONpP)

**Figure 1:**
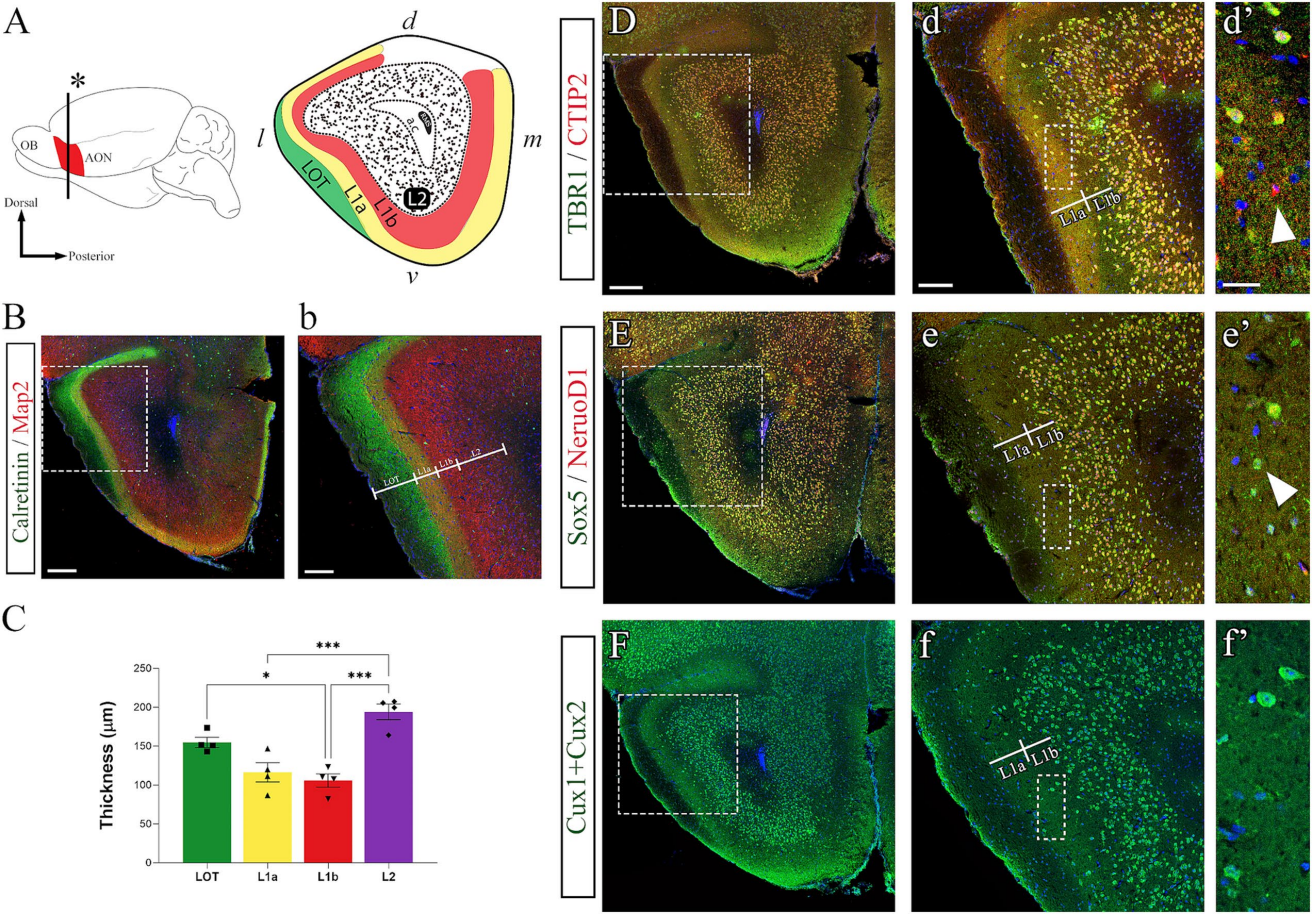
Molecular characterization of the adult AON. **(A)** Illustrations showing the anatomical location of the AON in the olfactory peduncle and a coronal section of the AON layers and anatomical features across the cardinal axes. **(B,b)** Immunohistochemistry to detect Calretinin (green) that labels LOT axons and Map2 (red) that labels apical dendrites from AON pyramidal neurons. Nuclei counterstained with Dapi (blue). Layer 1 is subdivided into layer 1a, where LOT axons synapse with apical dendrites (yellow), and layer 1b that is labeled only by apical dendrites (red). Layer 2 is a densely packed cell layer that is labeled with Map2 and Dapi (purple). **(C)** Quantification of the AON layers thicknesses (μm) measured at adult stages (postnatal day 21) from 4 animals (*n* = 4: males and females) with three independent samples. **(D–F)** Low magnification, **(d–f)** intermediate magnification, and **(d’–f’)** high magnification images representative of the molecular characterization of AON neurons using cortical markers for glutamatergic neocortical layer VI neurons (Tbr1 and NeuroD1), neocortical layer V (Ctip2), neocortical layers V-VI (Sox5), and neocortical layers II-IV (Cux1 + Cux2). **(d’-f’)** Differences in cortical marker expression in layer 1. Some neurons were detected expressing only Ctip2 (**d’**, arrowhead) or Sox5 (**e’**, arrowhead). Animals used were 8–10 weeks old. *d*, dorsal; *l*, lateral; *m*, medial; *v*, ventral; a.c., anterior commissure; LOT, lateral olfactory tract; RMS, rostral migratory stream. Statistical significance: *p < 0.05; ***p < 0.001. Scales bars: 200 μm in **(B,D)**; 100 μm in **(b’,d)**; 25 μm in **(d’)**.

As a laminated structure, the AONpP compartmentalizes its activity by segregating its axons, dendrites, and cell bodies in 2 layers delineated laterally by the LOT (Figure 1A) (Haberly and Price, 1978; Brunjes et al., 2005). We focused on the region of the AONpP where all of the layers were visible and identified by immunohistochemistry using calretinin (CR) to labels LOT axons and Microtubule associated protein-2 (Map2) to label the neuronal perikarya and dendrites. In the adult, LOT axons were exclusively labeled with CR (green) forming a layer with an average thickness of 156 ± 13 μm (Figures 1B,C) similar to the anterior piriform cortex (aPC) (Sarma et al., 2011; Martin-Lopez et al., 2019a). In L1a, LOT axons (CR^+^) make synapses with apical dendrites (Map2^+^) from L2 neurons, showing this sublayer (yellow) with a thickness of 116 ± 25 μm, thinner than that of the aPC (Figures 1B,C) (Sarma et al., 2011; Martin-Lopez et al., 2019a). L1b has no LOT axons and was only labeled with Map2 (red), displaying a thickness of 106 ± 17 μm (Figures 1A–C). The similar thickness of L1a and L1b in the AON resembled that seen in the posterior PC (pPC) (Martin-Lopez et al., 2019a) but contrasted the situation in aPC, where LIa is significantly thicker than LIb (Sarma et al., 2011; Martin-Lopez et al., 2019a). L2 displayed an average thickness of 194 ± 20 μm, significantly thicker than L1a and L1b (Figures 1B,C). Considering that the pPC is the associational system of PC (Luskin and Price, 1983b), these data suggested that the AON shares anatomical similarities with the pPC.

The cortical nature of the AON neurons was characterized at adult stages by IHC, using markers for transcription factors (TFs) typically expressed by neurons from different layers of the neocortex. Thus, Tbr1 (T-Box Brain Protein-1) was used to label glutamatergic neurons of pallial origin typically expressed by layer VI cortical neurons (Hevner et al., 2001; Brunjes and Osterberg, 2015; Canovas et al., 2015); Ctip2 (COUP-TF Interacting Protein 2) is a marker for layer V cortical neurons (Arlotta et al., 2005; Flores et al., 2025); NeuroD1 (Neurogenic differentiation factor 1) labels generating glutamatergic neurons embryonically, but remains expressed afterwards to maintain neuronal fate post-mitotically (Hevner et al., 2006; Aprea et al., 2014; Singh et al., 2022); Sox5 (SRY-box transcription factor 5) is expressed by corticofugal neurons from the deep cortical layers V-VI (Kwan et al., 2008; Harb et al., 2022; Takemoto et al., 2023); and Cux1 and Cux2 (Cut-like homeobox 1 and 2) were used as markers for upper cortical layers II-IV (Nieto et al., 2004; Cubelos et al., 2010). Our results showed that almost all neurons in the AONpP expressed these markers in both layers (Figures 1D–F). Specifically, we found co-expression of Tbr1 and Ctip2 (Figures 1D,d) in all neurons from L2 as previously described (Brunjes and Osterberg, 2015), but interestingly also in most neurons from L1, where some cells were observed expressing only Ctip2 (Figure 1d’, arrowhead). Similarly, most neurons expressed Sox5 and NeuroD1 (Figures 1E), although a small fraction in L1 exclusively expressed Sox5 (Figure 1E’, arrowhead). Cux1 + Cux2 (Figures 1F–f’) was expressed uniformly in both layers of the AONpP. The wide expression of these TFs in L2 neurons was expected as pyramidal-projection neurons are known to be the principal neurons of this layer (Brunert et al., 2023). However, what was intriguing was the detection of these TFs in L1 neurons across all regions of the AONpP. L1 is a plexiform layer that lacks glutamatergic neurons but contains different subpopulations of interneurons (Kay and Brunjes, 2014; Schuman et al., 2019), therefore the expression of these TFs mostly typical from projection neurons was unexpected. An exception was made with Ctip2, whose expression we previously reported among horizontal cells from LI in PC (Martin-Lopez et al., 2024). Horizontal cells are a subpopulation of neurons that lie alongside the internal surface of the LOT in the AON and PC and are presumed to be inhibitory GABAergic interneurons (Kay and Brunjes, 2014; Shepherd et al., 2021). The expression of Ctip2 by both glutamatergic and GABAergic projection neurons of the neocortex and striatum was previously reported (Arlotta et al., 2008; Martin-Lopez et al., 2019b; Flores et al., 2025), so that its presence in L1 was anticipated. On the other hand, the presence of neurons co-expressing Tbr1 with Ctip2 was unexpected but their full characterization falls beyond the scope of this paper.

Since neurons of the AON share embryonic origin with interneurons of the OB and originate in the most rostral part of the LGE (Puelles et al., 2000; Garcia-Moreno et al., 2008; Guo et al., 2019), we looked at the expression of these TFs in the OB (Supplementary Figure 2). As expected, Tbr1 and Sox5 were restricted to the glutamatergic projection neurons, M/Tc, of pallial origin (Supplementary Figures 2A,B). Ctip2 and NeuroD1 were widely expressed throughout the OB by most interneurons within the glomerular and granule cell layers (Supplementary Figures 2A,B), while Cux1 + Cux2 was mostly restricted to periglomerular neurons (Supplementary Figure 2C). These results suggested that the role of these TFs in the olfactory system are more diverse than simply conferring layer identity to projection neurons as occurs in the neocortex. Therefore, we attributed the presence of Ctip2^+^/Tbr1^+^, Sox5^+^/NeuroD1^+^, and Cux1 + Cux2^+^ cells within L1 to neurons that were likely displaced glutamatergic neurons of unknown function in the AON.

### Onset of neuronal phenotypes during the embryonic development of the AONpP

**Figure 2:**
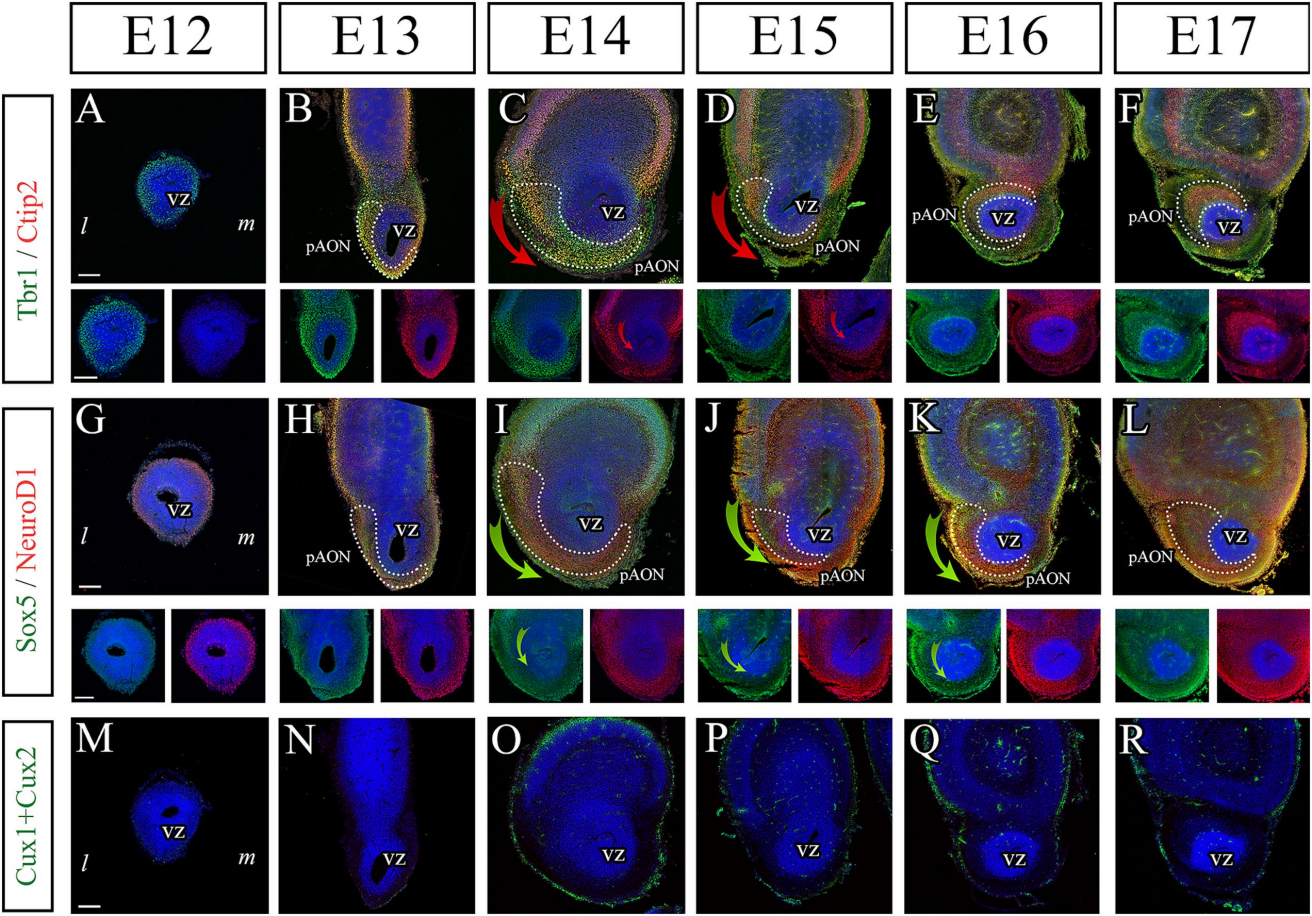
Onset of cortical neuronal markers in the developing AON. **(A–F)** Immunohistochemistry to detect Tbr1 (green) and Ctip2 (red) from E12 to E17 showing that only Tbr1 is expressed surrounding the VZ at E12 while Ctip2 becomes visible at E13. From E14 onwards there is an increase in Ctip2 expression in superficial regions colocalizing with Tbr1, suggesting neuronal differentiation. Ctip2 is seen following a dorsal-ventral pattern (**C,D**, red arrowheads) suggesting a role in differentiation across this axis. High magnification split channel images below corresponding low magnification images highlight difference in expression of Tbr1 and Ctip2. **(G–L)** Characterization of Sox5 (green) and NeuroD1 (red) during development of the AON. At E12 only NeurD1 is seen around the VZ, similar to Tbr1. From E13 onwards, Sox5 begins to be expressed by those cells migrating toward the surface of the developing AON, suggesting a role in later stages of maturation. Of interest is the dorsal-ventral gradient of Sox5 expression throughout development. High magnification split channel images below corresponding low magnification images highlight difference in expression of Sox5 and NeuroD1. As occurs with Ctip2, Sox5 also follows a dorsal-ventral expression pattern (**I–K**, green arrows). **(M–R)** Immunohistochemistry against Cux1 + Cux2 showing an absence of expression at all embryonic stages. **(A–L)** White dotted lines outline the pAON. In all images nuclei are counterstained with Dapi (blue). *l*, lateral; *m*, medial; VZ, ventricular zone; pAON, prospective AON. Scale bars: 200 μm.

Next, we studied the onset of these TFs during the development of the AONpP from E12 to E17. It should be noted that these TFs were previously reported to be expressed at embryonic stages during cortical development influencing neuronal maturation and differentiation (Nieto et al., 2004; Arlotta et al., 2005; Hevner et al., 2006; Kwan et al., 2008; Canovas et al., 2015; McKenna et al., 2015; Dennis et al., 2019; Flores et al., 2025). Tbr1, which is the most extensively studied marker during the differentiation of pallial projection neurons, was present as early as E12 in cells surrounding the ventricular zone (VZ) at the most rostral part of the telencephalic vesicle (Figure 2A). This is the region identified as the generative zone for the most rostral AON (AONpE) and the OB, while the main AONpP is generated from the most rostral LGE (Puelles et al., 2000; Garcia-Moreno et al., 2008; Huilgol and Tole, 2016). At E13 we found the first evidence of Ctip2 expression in the region belonging to the prospective AON (pAON), although it was seen in cells located distally to the VZ suggesting a role in neuronal differentiation as they migrated away from the VZ (Figure 2B, dotted line). From E14-E17, Tbr1 remained expressed in all AON neuroblasts, both those located proximal to the VZ and those that migrated toward the surface that make the main body of the AONpP, while Ctip2 was observed to be predominantly expressed only by those neurons located distally to the VZ (Figures 2C–F, dotted lines). Interestingly, Ctip2 expression was observed following a dorsal-ventral gradient (Figures 2C–F, red arrows). All Ctip2 cells also co-expressed Tbr1, which was considered indicative of AON neuronal maturation. Similarly, NeuroD1 was initially expressed by those cells surrounding the VZ at E12, likely involved on the first step of the neuronal determination in the AON (Figure 2G). During the following embryonic stages, NeurD1 was expressed by all neuroblasts in the pAON (Figures 2H–L). On the contrary, Sox5 followed a similar pattern to that from Ctip2, being first expressed at E13 distally to the VZ and following a dorsal to ventral gradient (Figures 2H–L, green arrows). This suggested that Sox5 was expressed by those neurons in advanced stages of differentiation and, as occurred with Ctip2, suggested a role in the dorsal-ventral axis differentiation (Figures 2H–L, dotted lines). Cux1 + Cux2 was not expressed embryonically (Figures 2M–R).

**Figure 3:**
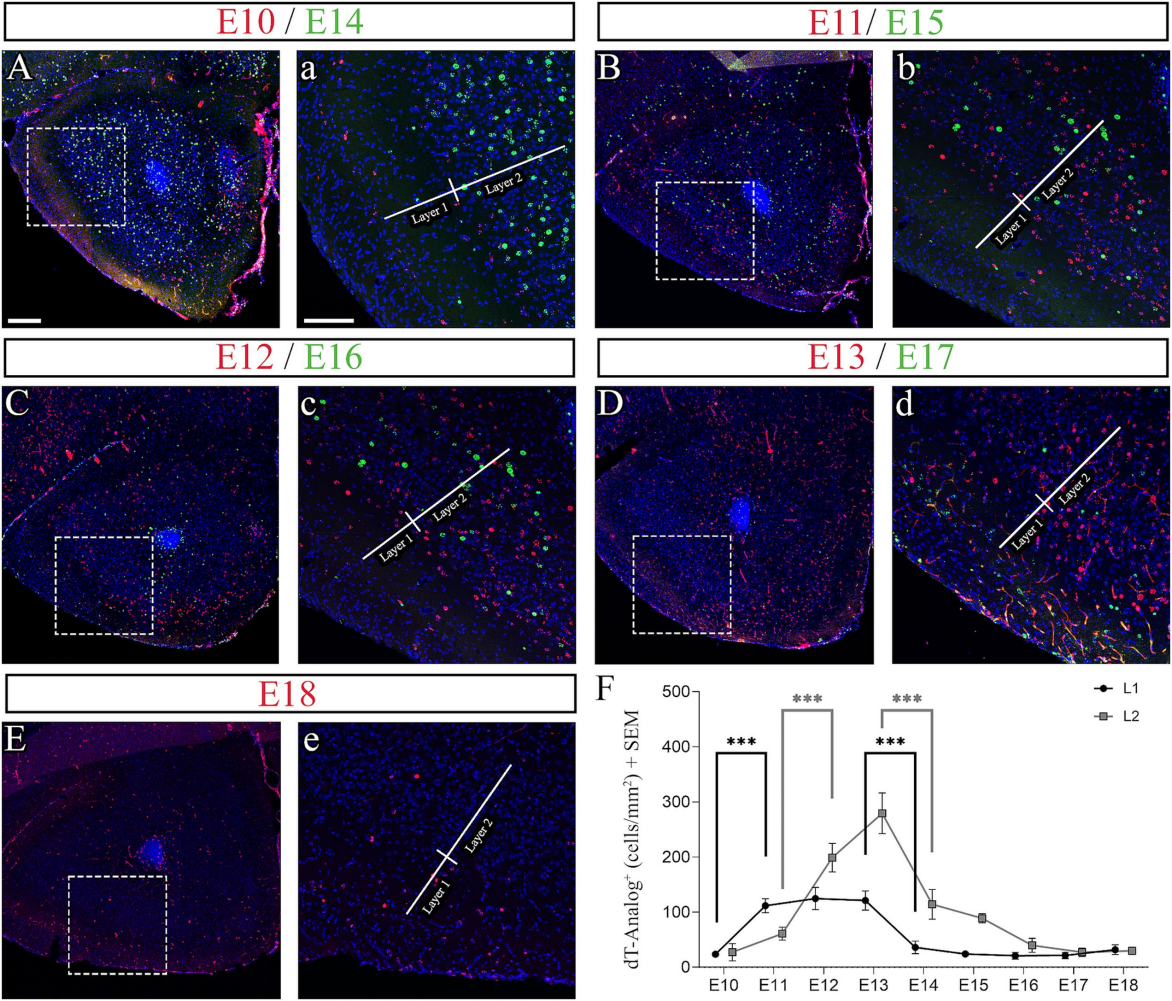
Neurogenesis of layers 1 (L1) and 2 (L2) of the adult AON studied following embryonic thymidine analog injections. **(A–E)** Low magnification and **(A–E)** high magnification images of immunohistochemistry to detect the thymidine analogs IdU (red) and CldU (green) with nuclei counterstained with Dapi (blue). L1 and L2 are highlighted with lines in **(a–e)**. **(F)** Quantification of IdU/CldU labeling in L1 and L2 separately per mm^2^. Cells were quantified from 6 animals at each timepoint (*n* = 6) with three independent samples. L1 shows a statistically significant increase in the number of cells between E10-E11 and a significant decrease between E13-E14. L2 shows a statistically significant increase in cells between E11-E12 and a significant decrease between E13-E14. Statistical significance: ***p < 0.001. Scale bars: 200 μm in **(A–E)**; 100 μm in **(a–e)**.

### Neurogenesis across the AONpP layers

We further studied neurogenesis of the AONpP by injecting pregnant females with thymidine analogs during the embryonic stages of E10-E18. This time window covers the period of differentiation of AON progenitor cells from the first postmitotic neuroblasts at E10 to the last day of embryonic development at E18 (Bulfone et al., 1995; Greig et al., 2013). Comparisons by age and layers are shown as combined data between males and females after we determined there were no statistically significant differences between the sexes (Supplementary Figure 1). Our data showed that neurogenesis in L1 began at E10 and increased significantly at E11 (Figure 3 and Table 2). From E11 to E13, neuronal generation plateaued producing 69.4% of the total number of cells in this layer. At E14, neurogenesis declined significantly through E18 (Figures 3A–F). This time window coincides with that seen in LI of PC (Martin-Lopez et al., 2019a). In contrast, neurogenesis in L2 showed an extended generation window that began at E10 but significantly increased at E12 (Figure 3F and Table 2), peaked at E13, abruptly declined at E14, and slowly decreased by E15 and E16 through the remainder of development (Figures 3A–F). The E11-E15 developmental window produced 85.8% of the total number of cells in L2, and resembled previous studies for this layer in PC, except the peak at E13 (Martin-Lopez et al., 2019a). Using immunohistochemistry to detect glial cells, we observed that none of the cells generated in L1 and L2 expressed the glial marker for astrocytes GFAP (Glial fibrillary acidic protein) nor the marker for microglia IBA1 (Ionized calcium binding adaptor molecule 1), concluding that none of the early cells in L1 and L2 were glial (data not shown).

**Figure 4:**
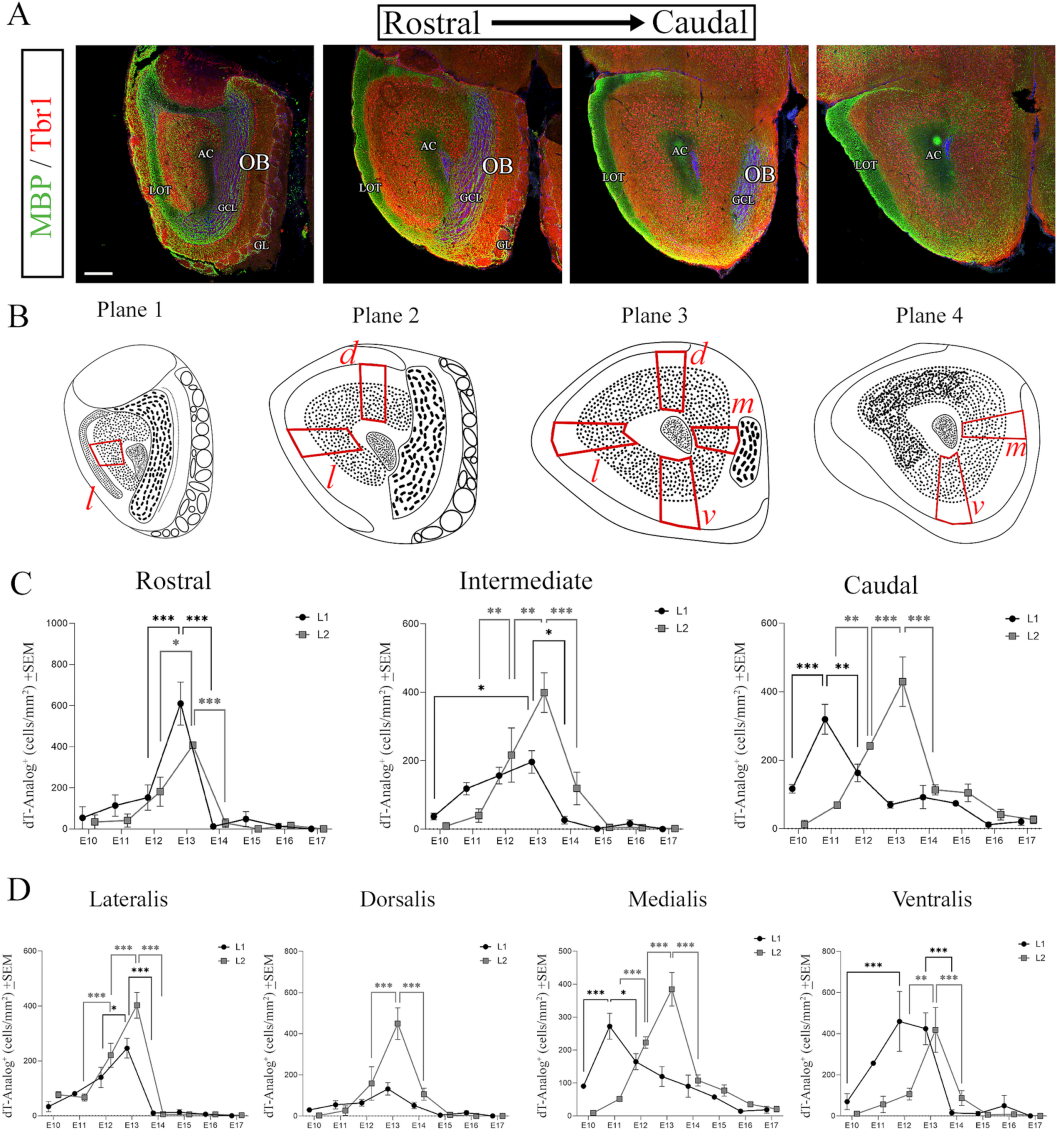
Neurogenesis of the AON studied along the rostral-caudal and cardinal axes. **(A)** Immunohistochemistry along the rostro-caudal axis to detect MBP (green) that labels myelin, and Tbr1 (red) that labels pallial projection neurons, with nuclei counterstained with Dapi (blue). **(B)** Illustration showing anatomical regions that divide the AONpP into four planes along the rostro-caudal axis using anatomical references from the MBP/Tbr1 staining. The location of the cardinal planes, highlighted in red (*l*, *d*, *m*, *v*), were used for quantification. **(C)** Quantification of analogs of thymidine labeled nuclei after injections embryonically (E10-E17) in both L1 and L2 along the rostro-caudal axis: Rostral counts come from Plane 1; Intermediate counts come from Planes 2 and 3; Caudal counts come from Plane 4. A caudal-to-rostral maturation gradient is seen only in L1. **(D)** Quantification of nuclei labeled with analogs of thymidine injected between E10-E17 in both L1 and L2 along the cardinal axes. Pars lateralis shows a peak of neurogenesis at E13 in L1 and L2, that increases and decreases significantly compared to E12 and E14. Pars dorsalis shows similar differences. Pars medialis shows a significant increase in the number of cells between E10-E11 in L1 and between E11-E12 and E12-E13 in L2. Pars ventralis shows significant increases in cells between E10-E13 in L1 and E12-E13 in L2. Numbers are condensed across the rostro-caudal axis on these comparisons. For all quantifications assessing the neurogenesis along the rostro-caudal axis and within the AONpP subsections a minimum of three animals (*n* = 3) were used at each timepoint with three independent samples. AC, anterior commissure; GCL, granule cell layer; GL, glomerular layer; LOT, lateral olfactory tract; OB, olfactory bulb; Pars *l*, pars lateralis; *d*, pars dorsalis; *m*, pars medialis; *v*, pars ventralis. Statistical significance: ***p < 0.001; **p < 0.01. Scale bars: 200 μm.

### AONpP neurogenesis along the cardinal axes

The importance of studying neurogenesis along the cardinal axes originated with previous studies in rats where AON neurogenesis exhibits different maturation gradients depending on the cardinal planes as well as along the caudal-to-rostral axis (Bayer, 1986a). In our work we tested to determine if these gradients also exist in the mouse AONpP. First, we used immunohistochemistry to label myelinated axons with myelin basic protein (MBP) and projection neurons using Tbr1. We used these molecular markers to establish planes along the rostro-caudal axis, and regions within these planes that were representative of the cardinal axes (Figures 4A,B). Along the rostro-caudal axis, we established four planes: plane 1 representative of the rostral region; planes 2 and 3 representative of intermediate regions; and plane 4 representative of caudal regions (Figures 4A,B). The distinction between planes was achieved by analyzing anatomical references highlighted by the MBP and Tbr1 staining. The rostral plane showed the most caudal portion of the OB, where axons from mitral and tufted cells that were labeled with MBP intermingled with interneurons of the glomerular (GL) and granule cell (GCL) layers (Figure 4A). Laterally, these axons clustered forming the lateral olfactory tract (LOT), that remained in this position across the entire rostro-caudal axis. In the rostral plane, we established that the AONpP was located between the LOT laterally and the GCL medially (Figure 4A). For the intermediate planes we chose sections that contained L2 clearly visible with cells labeled for Tbr1, which were surrounded laterally by the LOT and medially by the most caudal portions of the OB (Figures 4A,B). The most caudal plane was determined by having no identifiable OB structures and showing L2 neurons surrounding the AC and RMS (Figures 4A,B). Caudal to this plane, L2 will gradually become thinner along the lateral side before it transitions into PC. Using these planes as anatomical references, we proceeded to separate the AONpP into four different cardinal planes as follows: *pars lateralis* (Figure 4B, *l* in planes 1–3), *dorsalis* (Figure 4B, *d* in planes 2–3), *medialis* (Figure 4B, *m* in planes 3–4) and *ventralis* (Figure 4B, *v* in planes 3–4).

First, we determined if there was a caudal-to-rostral neurogenic gradient as previously found in rats (Bayer, 1986a). Our data showed, neurogenesis in caudal regions occurred earlier than in rostral areas in L1. In the caudal planes, neurogenesis was significantly higher at E11 compared to E10 and E12 (Figure 4C, Caudal), while in the intermediate and rostral regions this increase occurred at E13 (Figure 4C, Rostral and Intermediate). These data showed a 2-day delay in the maturation of the rostral and intermediate regions compared to caudal regions that confirmed the existence of a caudal-to-rostral gradient. On the contrary, neurogenesis in L2 showed no neurogenic gradients and neuronal production significantly increased simultaneously in all three regions. We observed an initial onset of neurogenesis in L2 between E12 and E13 and a subsequent decrease between E13 to E14, with a peak in production at E13 in all planes (Figure 4C). These data agreed with prior observations in L2 of mice injected with tritiated thymidine (Creps, 1974).

Having established the rostro-caudal gradients we analyzed neurogenesis in detail across the four cardinal planes: *pars lateralis*, *dorsalis*, *medialis,* and *ventralis* and studied their neurogenic gradients. In L1, neurogenesis was first detected to rapidly increase between the E10-E11 window in the *medialis* and *ventralis* regions which are representative of caudal regions. In these subdivisions, neurogenesis peaked at E11 in *pars medialis* (Figure 4D, Medialis) and at E12-E13 (Figure 4D, Ventralis) in *pars ventralis*. *Pars dorsalis* contained a small fraction of the total number of cells counted in L1 (Figure 4D, Dorsalis). *Pars lateralis* received the majority of cells between E11-E13 (Figure 4D, Lateralis). These data highlighted the caudal-to-rostral gradient previously seen in L1 along this axis. On the other hand, L2 showed a neurogenic window that extended from E11-E15 that produced 85.8% of the total number of cells in this layer. In all L2 subdivisions we found an abrupt increase of cells starting at E12 that peaked at E13 (Figure 4D). These results indicated that neurons in L2 were generated homogeneously in the entire AONpP and showed no observable gradients across all four cardinal planes.

### Neuroblast migration into the AON from the rostral lateral ganglionic eminence (rLGE) using a radial glial scaffold

**Figure 5:**
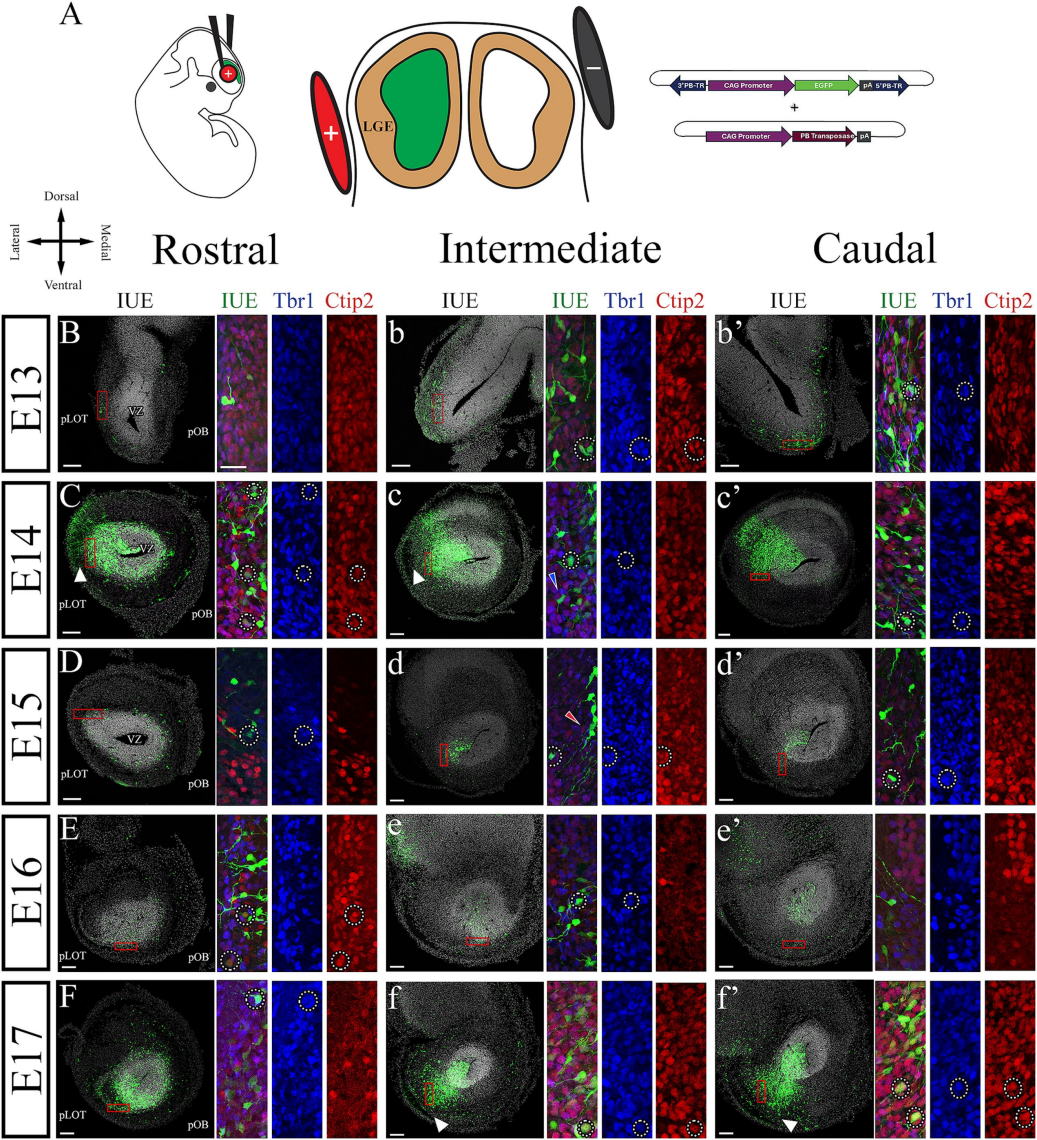
Migration and differentiation of neuroblasts targeting the AON from the rLGE. **(A)** Diagram showing the IUE technique and the plasmid mixture used to label AON progenitor cells. **(B–f’)** Immunohistochemistry of E13 to E17 coronal sections to detect Tbr1 (blue) and Ctip2 (red) in cells labeled by IUE at E11 (green). In all ages we observed colocalization of these markers with labeled neuroblasts (dotted circles). **(B–b’)** Representative images of the developing AON at E13 showing neuroblasts with multipolar-tangential migration with some differentiation into projection neurons in the intermediate and caudal regions (dotted circles). **(C–c’)** Images of the developing AON at E14 showing the neuroblasts migrating radially on caudal and intermediate sections, while they remain multipolar-tangential in the rostral region. Differentiation studied with Tbr1 and Ctip2 is now seen across all planes. pLOT is seen as a darken area in the lateral side of the sections. **(c)** Exemplary multipolar neuroblast labeled by IUE (red arrowhead). **(D–d’)** E15 sections of the developing AON showing a thicker L2 and radial migration and neuronal differentiation across all planes. **(d)** Exemplary bipolar neuroblast labeled by IUE (blue arrowhead). **(E–e’)** E16 images of the developing AON where migrating neuroblasts are seen mostly radially oriented and their leading processes appear branched. **(F–f’)** Representative images of the developing AON at E17 showing a massive influx of neuroblasts migrating from the VZ toward L2 using radial migration. At E17 many neurons were seen expressing Tbr1 and Ctip2 (dotted circles). rLGE, rostral lateral ganglionic eminence; IUE, in utero electroporation; pLOT, prospective lateral olfactory tract; pOB, prospective olfactory bulb; VZ, ventricular zone. Scale bars: 100 μm.

Finally, we used IUE to label AON neuroblasts and track their migration from the dorsal (pallial), most rostral portion of the LGE (rLGE). The rLGE is suggested to be the origin of all AONpP neurons (Puelles et al., 2000; Garcia-Moreno et al., 2008; Huilgol and Tole, 2016). AON progenitors were labeled at E11 using the piggyBac transposon expressing EGFP (Figure 5A), and embryos were collected from E13 to E17. Neuroblast migrations were studied along the rostro-caudal axis and their differentiation was analyzed by immunohistochemistry using Tbr1 and Ctip2 markers. The rLGE is located caudally to the developing AON, so that this structure was not represented in Figure 5, although the lateral VZ shown in these images were likely the rostral extension of the rLGE.

**Figure 6:**
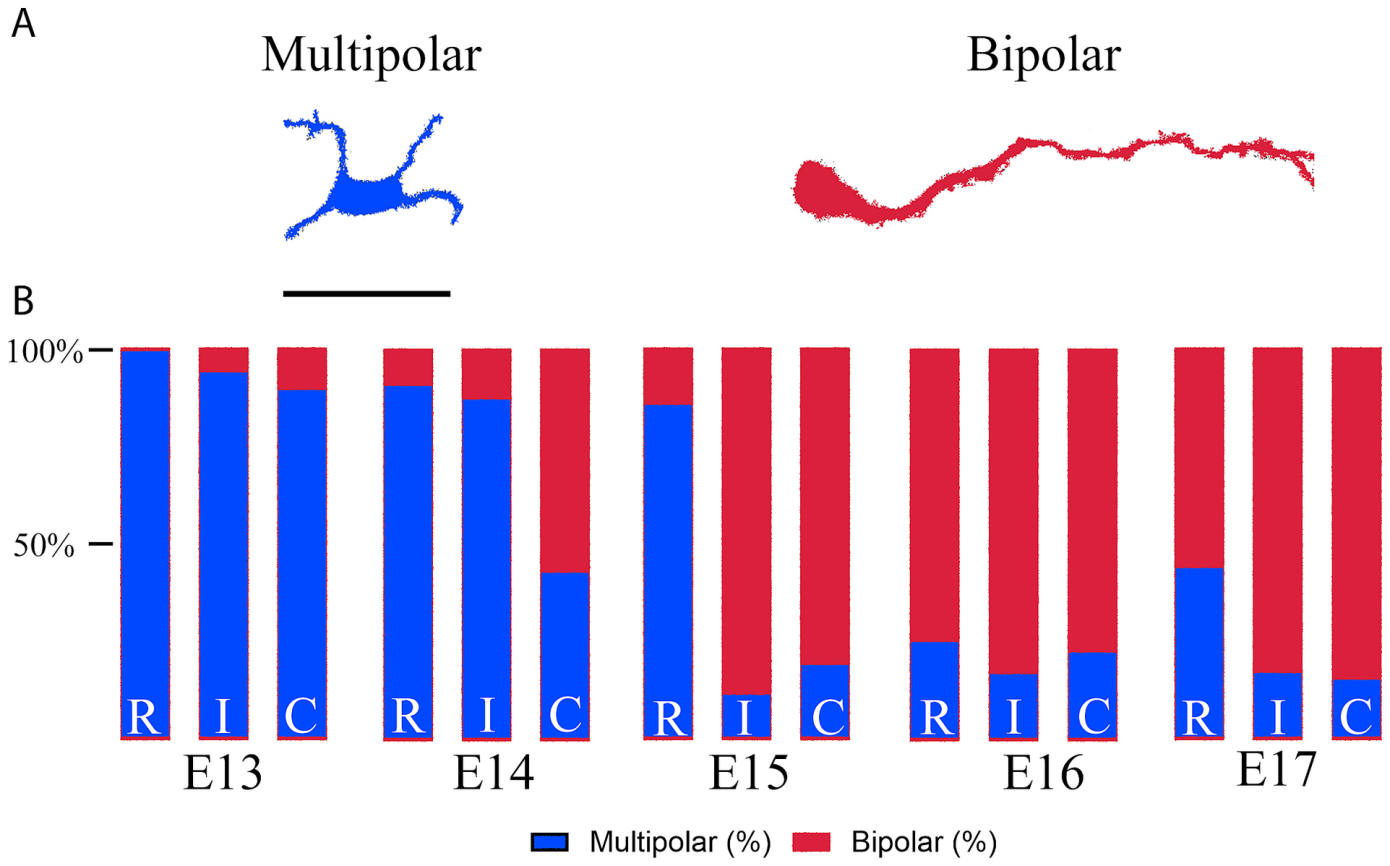
Morphological changes in migratory neuroblasts. **(A)** Illustration showing the morphological differences between multipolar and bipolar neuroblasts as used for this quantification. **(B)** Quantification of IUE labeled bipolar and multipolar neuroblasts within the prospective AON, expressed as percentages of a whole. R, rostral; I, intermediate; C, caudal. Scale bar: **(A)** 25 μm.

**Figure 7:**
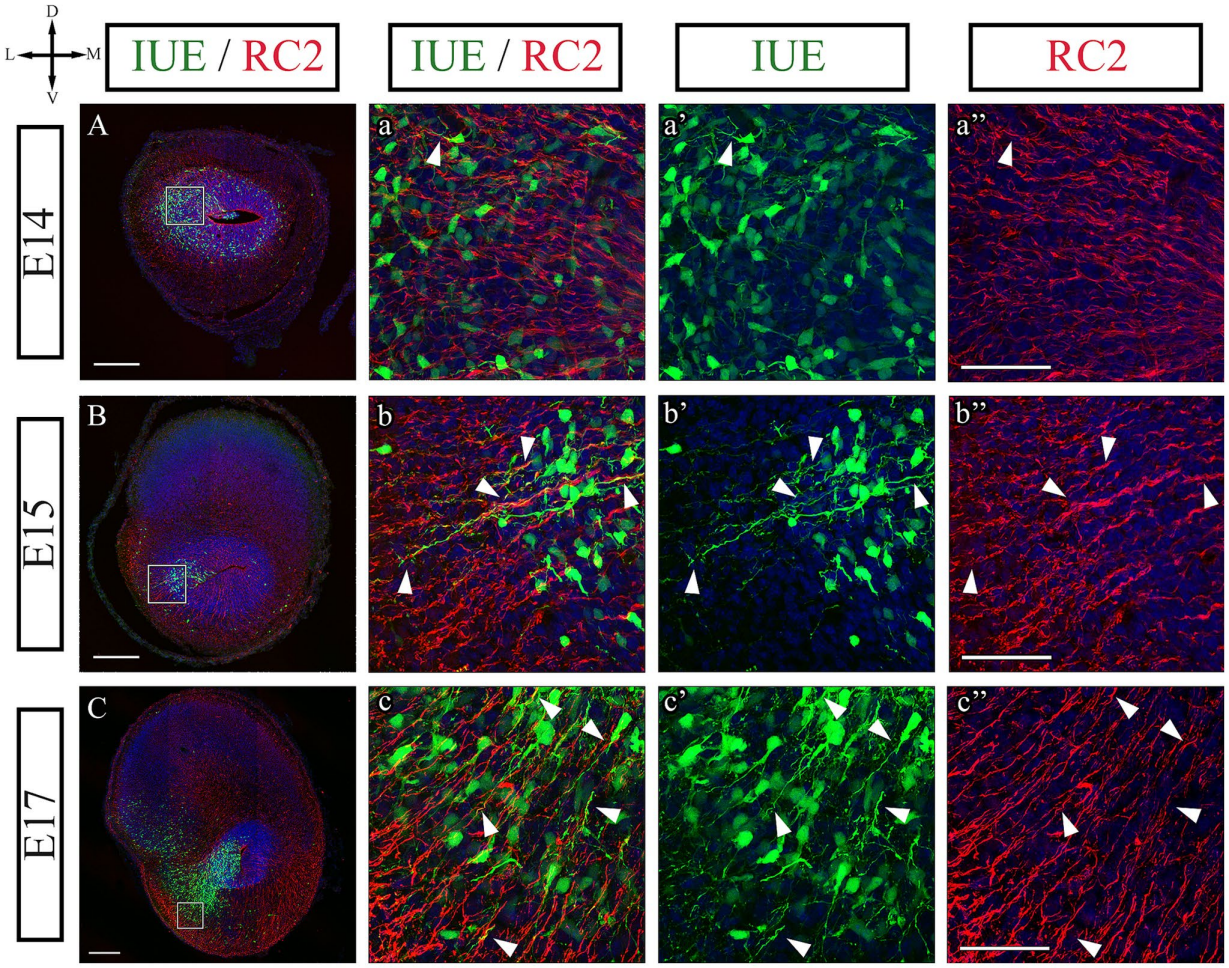
Radial glial scaffold for neuroblast migration. **(A–c”)** Immunohistochemistry of E14, E15, and E17 coronal sections to detect the radial glial cells marker RC2 (red) in combination with AON progenitor cells labeled with the piggyBac transposon by IUE into the rLGE (green). Nuclei counterstained with DAPI (blue). At all ages we observe proximity of RC2 labeled radial glial scaffolding with IUE neuroblast processes (white arrows), indicative of radial migration. **(A–a”)** Low and high magnification images of migrating neuroblasts and radial glial scaffolds at E14. At this age most neuroblasts are observed migrating tangentially to the radial glial scaffold, with some turning their leading processes into the radial scaffold from caudal regions (arrowheads). **(B–b”)** Low and high magnification images at E15 where more prominent neuroblast processes are found aligning with radial glia (arrowheads). **(C–c”)** Low and high magnification images at E17 where most migrating neuroblasts are radially arranged with the radial glial scaffold (arrowheads). AON, Anterior Olfactory Nucleus; IUE, in-utero electroporation; rLGE, rostral lateral ganglionic eminence. Scale bars: **(A–C)** 200 μm; **(a”–c”)** 50 μm.

At E13, most cells produced in the rLGE migrated tangentially to the ventricular zone (VZ) toward the prospective AON in all planes along the rostral-to-caudal axis (Figures 5B–b’). We noticed that neuroblasts were more numerous in the caudal regions compared to the intermediate and rostral planes (Figures 5B–b’). This finding suggests the existence of a caudal-to-rostral migratory gradient that resembles that seen in the PC (Martin-Lopez et al., 2019a). This gradient should not be confused with the caudal-to-rostral neurogenic gradient determined in L1 with the injections of thymidine analogs (Figure 4). At this age, some labeled neuroblasts expressed both Tbr1 and Ctip2 markers in the caudal and intermediate regions (Figures 5B–b’, dotted circles). We estimated the percentage of cells expressing one, both, or neither of these TFs in cells expressing the EGFP from the IUE, showing the caudal-to-rostral trend on neuronal maturation (Supplementary Figure 3). However, due to the heterogeneity of the IUE labeling between the different animals, it is important to note that these quantifications only represent an approximation of the real situation. Homogeneous IUE fields are elusive in embryos but would be required in multiple animals to confirm the differentiation dynamics of rLGE neuroblasts migrating to the pAON. At E14 we obtained some embryos with wide electroporations that extended the labeling from the rLGE to the AON-VZ (Figures 5C–c’). This extensive labeling allowed us to simultaneously observe neuroblasts migrating from the rLGE (Figures 5C–c’, red rectangles) and the neuroepithelial cells forming the VZ of the AON primordium (Figures 5C–c’, VZ). The migrating neuroblasts extended leading processes for migration and showed an orientation predominantly tangential to the VZ (Figures 5C–c, blue arrowheads). At this age some neuroblasts expressed both Tbr1 and Ctip2 (Figures 5C–c’, dotted circles; Supplementary Figure 3). In these animals we also observed thin cellular processes extending from the VZ that reached the surface of the brain tissue (Figures 5C–c’, white arrowheads), which presumably belonged to radial glial processes expressing RC2 (see below). Radial glial processes are well known to be necessary in neo- and paleo-cortices to support neuroblast migration toward their final destination (Rakic, 2003). Notably at E14, only neuroblasts located in the caudal regions showed bipolar cells extending an elongated leading process, compared to those from the intermediate and rostral regions that where multipolar were predominate (Figures 5c,c’, 6, 7A–a’’). Changes between multipolar to bipolar morphologies is characteristic of neuronal differentiation in the cortex after mitosis, which is directly related to a transition between tangential to radial migration (Cooper, 2014). This transition in the migratory pattern was more evident at later embryonic ages, where the radial orientation of neuroblasts were seen more rostrally into the intermediate regions at E15 (Figures 5d,d’) and across all regions at E16-E17 (Figures 5E–f’, arrowheads). To provide a better picture of this migratory transition from multipolar to bipolar patterns across the rostral-caudal axis, we estimated the percentage of neuroblasts that exhibited these morphologies (Figure 6). As seen here, there was an evident transition of morphologies from multipolar to bipolar that followed a caudal-to-rostral gradient across all embryonic ages, with a clear transition to be mostly bipolar at E15 (Figures 6A,B). As the migratory and maturation processes progressed and cells were moving away from the VZ toward the surface to from the prospective L2, the differentiation markers Tbr1 and Ctip2 increased their expression in EGFP-labeled cells. Different stages of differentiation were visible at E16 with cells expressing either Tbr1 or Ctip2, while at E17 multiple neurons were seen co-expressing Tbr1 and Ctip2 (Figures 5E–f’ and Supplementary Figure 3).

To confirm that the thin processes extending from the VZ were indeed radial glial cell processes, we stained the sections with the antibody RC2 that specifically recognizes radial glia cells (Hartfuss et al., 2001; Martin-Lopez et al., 2019b). Our results showed that all radial fibers extending from the VZ within the AON primordium expressed RC2, indicating they belonged to radial glial cells and fibers (Figure 7). These processes were used by AON neuroblasts to migrate radially throughout development (Figure 7, arrowheads). At E14 most neuroblasts were seen migrating tangentially to the radial glial processes, with some cells beginning to turn their leading processes toward the radial scaffold in caudal regions (Figures 7A–a”, arrowheads). At E15, more cells were observed aligning their processes to the radial glial scaffold establishing close contacts with those fibers in what it is known as radial migration (Figures 7B–b”, arrowheads; Figure 6B). At E17, the end of embryonic development, most migrating neuroblasts were observed establishing close contact with the radial processes and migrate radially (Figures 7C–c” arrowheads). Since the use of radial glia processes is a requisite for the migration of projection neurons in the brain (Rakic, 2003), our results experimentally confirm the cortical nature of the AON.

Collectively, our data demonstrated that AON neuroblasts followed a caudal-to-rostral migratory (not neurogenic) gradient. Beginning in caudal regions at E14 and continued through the entirety of embryonic development, neuroblasts from AON progenitor cells used a radial glia scaffold to migrate, transitioning from multipolar to bipolar morphologies. While these neurons continued to migrate toward the developing AON, they began to express Tbr1 and Ctip2 across all ages of embryonic development, indicating their determination to become AON projection neurons, or in the case of Ctip2 horizontal interneurons from L1, prior to reaching their final destination.

## Discussion

The AON is a processing center where olfactory information is sorted and compared between both hemispheres of the brain via heavy interconnections with both OBs and other areas of the brain. These extensive interhemispheric connections then impact social behaviors such as fear responses or memory formation related to smell (Brunjes et al., 2005; Brunert et al., 2023). Although critically important to understand its function, developmentally the AON is not well characterized and has not yet benefited from the current methods of cell labeling and tracking, particularly at embryonic stages (Bayer, 1986a; Brown and Brunjes, 1990; Marchand and Belanger, 1991; Armstrong and Brunjes, 1997; Brunjes et al., 2014; Brunjes and Osterberg, 2015; Collins and Brunjes, 2020). In this work, we employed different approaches to study embryonic tissues—including injections of thymidine analogs, immunohistochemistry, and labeling of embryonic progenitors using the piggyBac transposon inserted by IUE - to analyze the embryonic development of the AON and study its neurogenesis, phenotypical characterization, neuroblast migration, and neuronal differentiation.

We found wide expression of TFs in most cells of the AON. These TFs were selected for testing because they have been found to have specific roles in establishing laminar identity in cortical neurons during development, making their expression in the AON intriguing. For example, Tbr1 is known to be necessary for the embryonic determination of all pallial (= cortical) glutamatergic neurons in neo- and paleo- cortex, and to then gradually decline its expression to regulate the identity and connectivity of layer VI neurons in the neocortex (Hevner et al., 2001; Garcia-Moreno et al., 2008; Fazel Darbandi et al., 2018; Co et al., 2022). The identity of layer VI neurons is then maintained by repressing the expression of Ctip2, Fezf2, and Sox5, which are overexpressed in layer V (Bedogni et al., 2010; Canovas et al., 2015; Harb et al., 2022). The high levels of these TFs in layer V are thought to be maintained by the AT-rich sequence binding protein 2 (Satb2) (McKenna et al., 2015), which also acts in upper cortical layers (II-IV), together with other TFs such as COUP-TF1 and FOXG1, to determine layer identity through the expression of Cux1 and Cux2 (Armentano et al., 2007; Cubelos et al., 2015; Ba et al., 2023). However, in PC (part of paleocortex) these TFs are expressed across the 3 laminae but surprisingly in a reverse order to that seen in neocortex (Diodato et al., 2016). Tbr1 is expressed across all layers of PC (IIa, IIb and III), whereas Ctip2 is overexpressed superficially in layer IIa, and Cux1 and Brn1 are overexpressed in the deeper layers IIb and III - all of which seems to establish layer identity (Brunjes and Osterberg, 2015; Diodato et al., 2016; Nasu et al., 2020). This may explain the reverse “outside-in” neurogenic gradient that we reported within LII (a and b) of PC (Martin-Lopez et al., 2019a). Surprisingly, none of these laminar patterns were seen in neurons of the AON (Figure 1 and Supplementary Figure 1) even though it shares its developmental origin with PC in the LGE (Huilgol and Tole, 2016). Due to the complexity of these molecular pathways, that are still incomplete and not well understood, it is premature to interpret the ubiquitous expression of these TFs in AON cells. We can speculate with the hypothesis that the AON is an ancient cortex which expanded during evolution into the PC and later neocortex, which perhaps conferred different roles to these TF as the cortex segregated into distinct layers with stereotyped neuronal and synaptic circuits.

Our study did not show a caudal-to-rostral neurogenic gradient in L2 of the AON, which aligns with our previous data in PC (Martin-Lopez et al., 2019a) and contrasts with descriptions in rats (Bayer, 1986a, 1986b). Although Creps (1974) previously reported data similar to ours in mice, we found sharply different neurogenic gradients across the cardinal axes. While both Creps and we found that *pars lateralis* has a sharp increase in neurogenesis at E13 (Creps, 1974), Creps found an elongated neurogenic window in the *pars dorsalis* and *pars lateralis* that was also seen by Bayer in rats that we could not detect (Bayer, 1986a). It seems reasonable to speculate that the differences between our data could be the result of sampling or the age of study, as well as the advances that have occurred in strategies for labeling cells. Furthermore, if the caudal-to-rostral gradient that we observed in L1 also occurs in rats remains open for further investigation.

As a cortical structure, the AON communicates with other regions of the brain predominantly in L1. In the adjacent PC, LI is subdivided into two distinctive sublayers that compartmentalize the information received directly from the OB (LIa) versus that forming associational cortico-cortical connections (LIb). Sublayers 1a and 1b have been also reported to play similar roles in the AON (Brunert et al., 2023) but their thicknesses have yet to be measured. Previously in PC, the width of sublayers Ia and Ib were used to determine how much of a primary or associational cortex PC was along its rostral to caudal axis (Luskin and Price, 1983b; Bekkers and Suzuki, 2013). For instance, the anterior region of PC (aPC) has a thicker LIa pointing to its role as a primary cortex. However, pPC has a thicker LIb indicating that it has increased associational connectivity and is predominantly a secondary cortex (Martin-Lopez et al., 2019a). Since the AON showed that L1b had a similar thickness (Figure 1) to that of LIb in pPC (Martin-Lopez et al., 2019a), we speculate their functions are similar and therefore, the AON could have a predominant role as an associative cortical structure (Luskin and Price, 1983a; Luskin and Price, 1983b; Schwob and Price, 1984). These observations reinforce the idea that the AON behaves more as a secondary rather than a primary olfactory cortex, as could be inferred by the extraordinary connectivity that the AON has with many other regions of the brain (Brunjes et al., 2005; Lei et al., 2006; Yan et al., 2008; Brunert et al., 2023). Determining the significance of the primary olfactory functions compared with the associational processing of olfactory information within the AON remains unknown.

This paper is the first in which AON progenitor neurons were studied during development isolated from other olfactory regions that differentiate from the rostral telencephalon, such as the OB and PC (Huilgol and Tole, 2016). Here, we used IUE to precisely target the dorsal, most rostral part of the LGE (rLGE) to study neuroblast migration and differentiation during the embryonic development of the AON. This allows for the investigation of the similarities and differences of AON development compared to adjacent regions: the OB rostrally and the aPC caudally. With our method using the piggyBac transposon and IUE, we were able to target the source of most projection neurons and likely some interneurons such as horizontal cells from L1. However, Garcia-Moreno reported that while regions of the rostral telencephalon that give rise to the aPC, TuS and the OB receive cells from the rLGE, there are also other areas that produce neurons that target olfactory regions such as the rostromedial telencephalic wall (RMTW) - a region located dorsally to the septum in the embryo (Garcia-Moreno et al., 2008). Therefore, it is reasonable to consider that the AON would also receive at least small subpopulations of neurons from the RMTW. Demonstration of which regions of the rostral telencephalon produce neurons migrating to the AON is a technical challenge, therefore this question remains to be fully characterized.

In summary, this work presents a new and detailed map of AON development that contributes with the following findings: (1) AON neurons from L1 and L2 express most of the common cortical neuronal markers: Tbr1, Ctip2, Sox5, NeuroD1, and Cux1 + Cux2 at adult stages without showing any laminar distribution. (2) Embryonically, Tbr1 and NeuroD1 are first expressed in neuroblasts next to the VZ while differentiating to become projection neurons. As they locate distally from the VZ and move superficially, these neuroblasts begin to co-express Ctip2 and Sox5. Cux1 + Cux2 is not expressed embryonically suggesting a role in postnatal differentiation. (3) Neurogenesis in the AON occurs in the E11-E13 developmental window in L1, following a caudal-to-rostral neurogenic gradient. In L2, neurogenesis is delayed 1 day to the E12-E14 developmental window, peaking at E13 with no gradients. (4) Across the cardinal axes, L1 neurogenesis follows a medial-ventral-lateral progression of generation not seen by L2 neurons. (5) We confirm that the rLGE is the source of AON projection neurons. From here, AON neuroblasts transition from a multipolar to bipolar morphologies that relate to a change in migration from tangential to radial using a radial glial scaffold. In this process AON neuroblasts follow a caudal-to-rostral migratory and differentiation gradient.

## Data Availability

The original contributions presented in the study are included in the article/Supplementary material, further inquiries can be directed to the corresponding author.

## Supplementary material

Supplementary Figure 1 Neurogenesis comparison between males and females in the developing AON. Three males and three females were used in analysis (*n* = 3). **(A)** Quantification of IdU/CldU labeling in L1 between males and females from E10 to E18. **(B)** Quantification of IdU/CldU labeling in L2 between males and females from E10 to E18. In both layers, no statistical difference was found between males and females across all time points. Sex was collapsed into one group for further analysis of the developing AON.

Supplementary Figure 2 Expression of the transcription factors Tbr1 and Ctip2 in the adult OB. **(A)** Immunohistochemistry to detect Tbr1 (green) and Ctip2 (red) in the adult mouse OB. **(B)** Immunohistochemistry to detect Sox5 (green) and NeuroD1 (red) in the adult mouse OB. **(C)** Immunohistochemistry to detect Cux1+Cux2 (green) in the adult mouse OB. In all images, nuclei were counterstained with DAPI. GL, glomerular layer; EPL, external plexiform layer; MCL, mitral cell layer; IPL, internal plexiform layer; GCL, granule cell layer; RMS, rostral migratory stream. Scale bar: 100 μm.

Supplementary Figure 3 Percentage of Tbr1 and Ctip2 expression within migrating neuroblasts labeled with the piggyBac transposon. Quantification of the expression of Tbr1 and Ctip2 depicted as percentages of a whole comparing the expression of Tbr1, Ctip2, co-expression, and negative expression in IUE+ neuroblasts. IUE+, in-utero electroporation positive; R, rostral; I, intermediate; C, caudal.
