# Supplementary figures and images for: Laminar organization of the anterior olfactory nucleus—the interplay between neurogenesis timing and neuroblast migration
Source: Front Neurosci. 2025 Apr 30;19:1546397. doi: 10.3389/fnins.2025.1546397 (PMC12075217; doi:10.3389/fnins.2025.1546397)

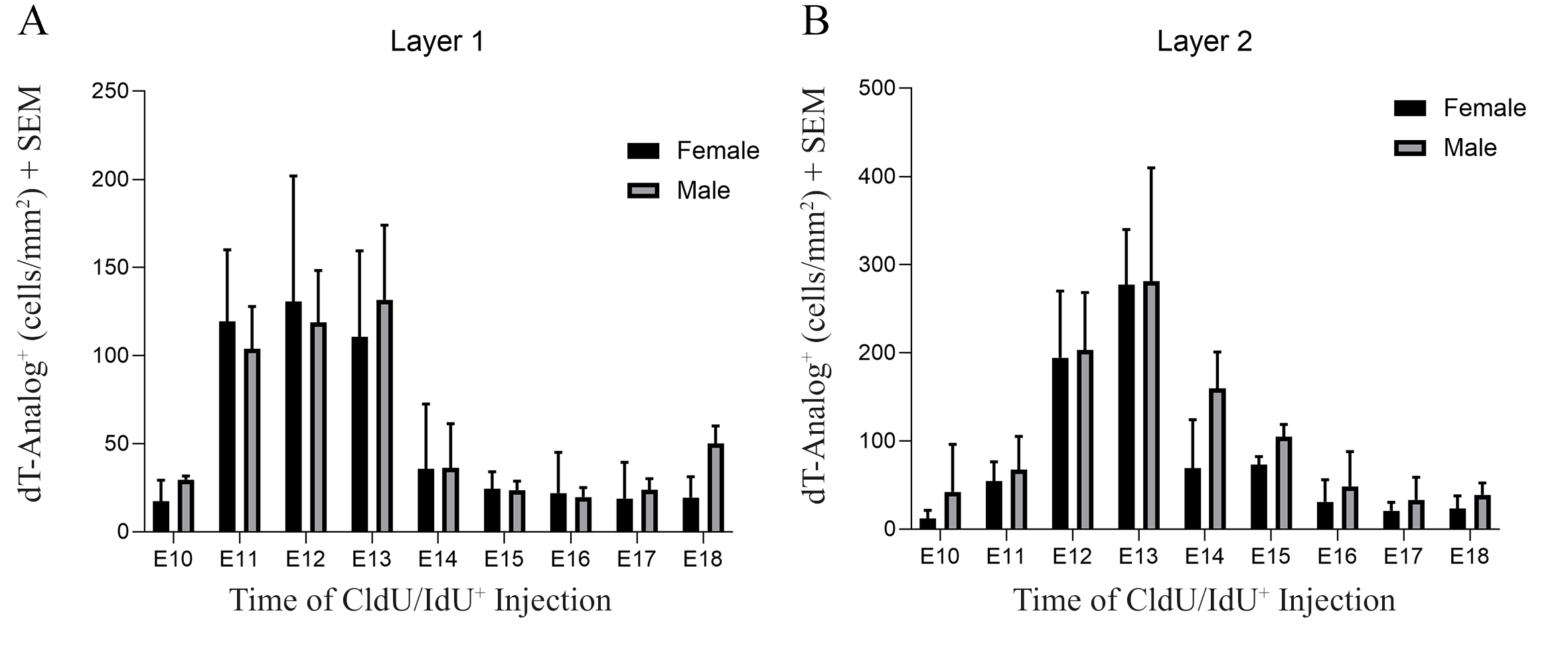

Supplement: Supplementary Figure 1 — Neurogenesis comparison between males and females in the developing AON. Three males and three females were used in analysis (n = 3). (A) Quantification of IdU/CldU labeling in L1 between males and females from E10 to E18. (B) Quantification of IdU/CldU labeling in L2 between males and females from E10 to E18. In both layers, no statistical difference was found between males and females across all time points. Sex was collapsed into one group for further analysis of the developing AON. [file Image_1.TIF]

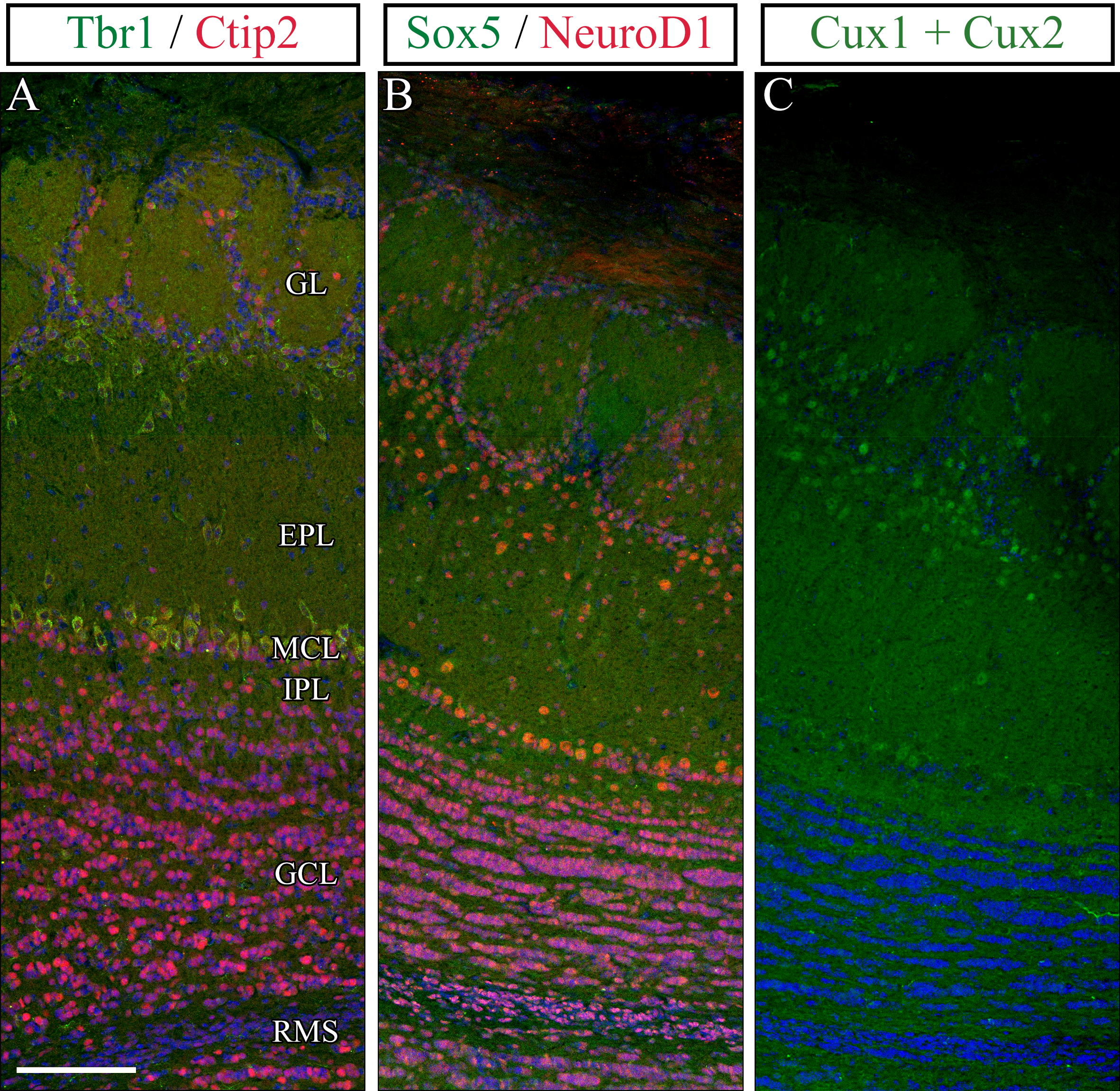

Supplement: Supplementary Figure 2 — Expression of the transcription factors Tbr1 and Ctip2 in the adult OB. (A) Immunohistochemistry to detect Tbr1 (green) and Ctip2 (red) in the adult mouse OB. (B) Immunohistochemistry to detect Sox5 (green) and NeuroD1 (red) in the adult mouse OB. (C) Immunohistochemistry to detect Cux1+Cux2 (green) in the adult mouse OB. In all images, nuclei were counterstained with DAPI. GL, glomerular layer; EPL, external plexiform layer; MCL, mitral cell layer; IPL, internal plexiform layer; GCL, granule cell layer; RMS, rostral migratory stream. Scale bar: 100 μm. [file Image_2.TIF]

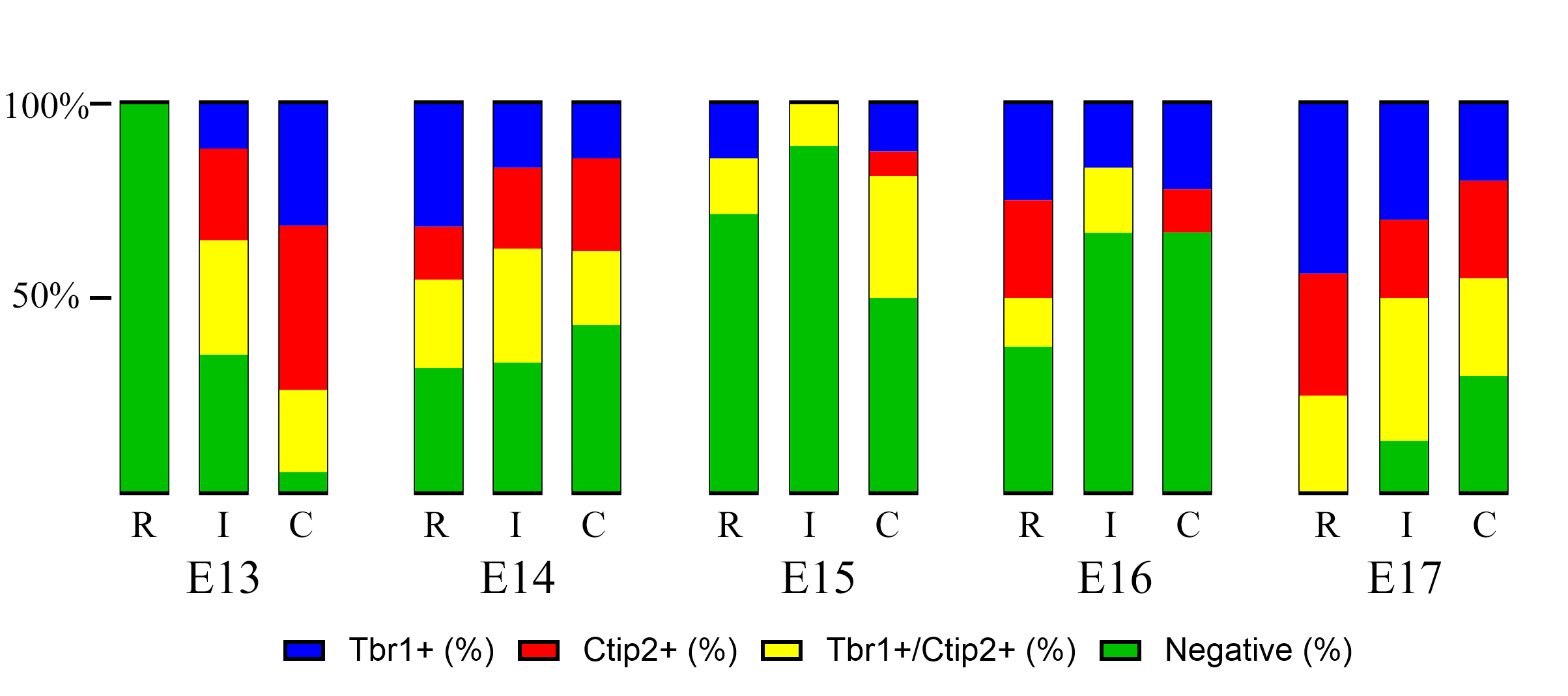

Supplement: Supplementary Figure 3 — Percentage of Tbr1 and Ctip2 expression within migrating neuroblasts labeled with the piggyBac transposon. Quantification of the expression of Tbr1 and Ctip2 depicted as percentages of a whole comparing the expression of Tbr1, Ctip2, co-expression, and negative expression in IUE+ neuroblasts. IUE+, in-utero electroporation positive; R, rostral; I, intermediate; C, caudal. [file Image_3.TIF]
